# Efficacy and tolerability of pharmacotherapy for post-stroke depression: a network meta-analysis

**DOI:** 10.18632/oncotarget.23891

**Published:** 2018-01-03

**Authors:** Linghui Deng, Shi Qiu, Yan Yang, Lu Wang, Yuxiao Li, Jing Lin, Qiang Wei, Lu Yang, Deren Wang, Ming Liu

**Affiliations:** ^1^ Stroke Clinical Research Unit, Department of Neurology, West China Hospital, Sichuan University, Chengdu, Sichuan, China; ^2^ Department of Urology, Institute of Urology, West China Hospital, Sichuan University, Chengdu, Sichuan, China; ^3^ Kidney Research Institute, Division of Nephrology, West China Hospital, Sichuan University, Chengdu, Sichuan, China

**Keywords:** post-stroke depression, pharmacotherapy, network meta-analysis, Hamilton depression scale

## Abstract

**Background:**

Post-stroke depression (PSD) occurs in approximately one third of stroke survivors, leading to great disability and mortality. As there is no consensus on the optimal pharmacological treatment for PSD, we aimed to evaluate the relative efficacy and tolerability of the available pharmacological interventions.

**Materials and Methods:**

We did a network meta-analysis to incorporate evidence from relevant trials providing direct and indirect comparisons. We searched PubMed, the Cochrane Library Central Register of Controlled Trials, Embase and the reference lists of relevant articles up to March, 2017 for randomized controlled trials (RCTs), for different pharmacotherapies of PSD. For efficacy analysis, the primary outcome was the mean change in Hamilton Depression Scale (HAMD) score between baseline and endpoint. For tolerability analysis, the outcome was presented by the discontinuation for any reason. This study is registered with PROSPERO, number CRD42016049049.

**Results:**

From a total of 869 citations, 15 RCTs with 876 participants were included. 13 drugs were considered. For efficacy, paroxetine ranked the best for HAMD reduction, followed by imipramine, reboxetine, nortriptyline, citalopram and fluoxetine at the end of treatment. However, duloxetine ranked the best at 4-week and 8-week duration for HAMD reduction. For tolerability, paroxetine ranked the best but there is no significant result between any comparisons.

**Conclusions:**

Paroxetine is probably the best option to consider for patients with PSD. To get a quicker relief of depression, duloxetine might be useful for its rapid onset of antidepressant action. The tolerability was comparable among all the antidepressants. But more high-quality RCTs are needed.

## INTRODUCTION

Stroke can be a life-threatening accident and one in which psychiatric sequelae throughout recovery are very common. Depression after cerebrovascular accidents is reported to affect 31% stroke survivors worldwide within 5 years after stroke onset according to a recent study [1]. Apart from being tormenting in and of itself, post-stroke depression (PSD) has been shown to be deleterious for patient outcome, including poor compliance with rehabilitation trainings, increased use of healthcare resources, poor physical and cognitive functional rehabilitation, higher recurrence rate of stroke, greater dependency and earlier mortality involving suicide [2–7].

Stroke-associated depression may have potential differences from general depression. The causal relationship between stroke and subsequent depressive disorder can be quite complicated for the following reasons. Firstly, the pathogenesis of PSD is in the dispute about whether it is directly due to cerebral impairments, or is a indirectly consequence of the negative psychological reaction to such a distressing cerebrovascular accident [8]. Many confounding factors such as apoplexy severity and lesion locations have been extensively investigated as risk factors for PSD [9]. Secondly, incidence of depression was higher in stroke survivors than general population [10], even higher than their counterparts with comparable physical impairments [11]. In return, evidence suggests that depression severity was an independent predictive factor of severity of impairment in daily activities among stroke survivals [12]. Judging by the possibility that PSD differs in underlying pathogenesis, it may be improper to extrapolate data of managements from general depression population to PSD patients. It is thereby crucial to develop proper effective antidepressant management for this specific population to moderate depression after stroke. Pharmacological interventions are regarded as a cornerstone of antidepressant treatment and there were few meta-analyses that have examined the effectiveness of antidepressants in treating PSD [13–16]. Within drug categories, heterocyclic antidepressants and selective serotonin reuptake inhibitors (SSRIs) were the best studied. The first two randomized controlled trials (RCTs) concerning treatment of PSD, published in 1984 and 1986 respectively, provided strong evidence that heterocyclic antidepressants were significantly more effective than placebo in reducing depression after stroke [16, 17]. However, unwanted side effects were observed in this classic antidepressants due to its affinity with muscarinic cholinergic and histaminergic receptors. In the light of the results of a meta-analysis [18], there is also strong evidence demonstrated a significant benefit of selective serotonin reuptake inhibitors (SSRIs) in managing depression. Other categories such as selective noradrenaline reuptake inhibitors (SNRIs) and monoaminergic drugs also showed effectiveness in treating PSD [19, 20]. Moreover, apart from effectiveness in treating depression, several types of antidepressants like SSRIs and tricyclic antidepressants (TCAs) were also proved to be associated with several beneficial impacts on recovery of physical, cognitive, and even neurological function in recovering stroke patients, probably owning to the drug-induced change of cortical excitability, anti-inflammatory effects, increased angiogenesis and hippocampal neurogenesis [19–22].

Though SSRIs are gaining popularity as first-line antidepressant treatment for geriatric population [23], there are no studies providing direct evidence to show a comprehensive superiority of SSRIs over TCAs in treating PSD, nor studies providing conclusive data to determine whether any specific drugs are superior to others [24]. Beside, conclusive evidence for the tolerability of antidepressants in PSD patients is lacking.

Taking these into consideration, we thereby performed a network meta-analysis (NMA) using literature searches to systematically assess and rank the effectiveness and tolerability of antidepressant agents in patients with PSD.

## RESULTS

### Search and selection

**Figure 1:**
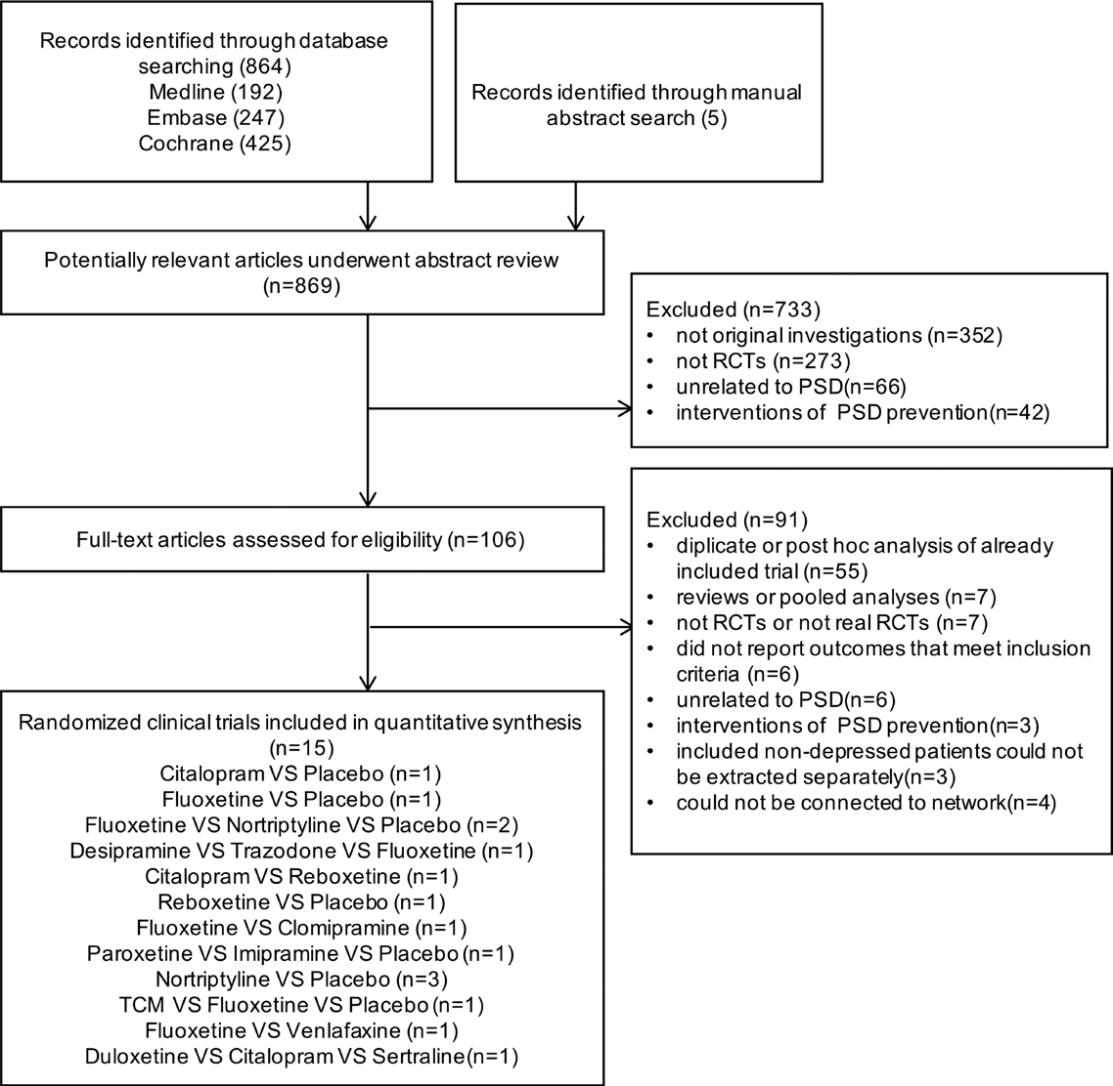
Flow chart of study identification and selection procedure

Of 869 citations identified through the search algorithm, 15 RCTs, including 876 participants were contained in this NMA (Appendix 3). The Systematic Reviews and Meta-analysis (PRISMA) flowchart depicting electronic searching processes is presented in Figure 1.

### Characteristics of studies and participants

The trials comparing 13 antidepressant agents were published between 1984 and 2012. Detailed study characteristics are given in Table 1. Eleven (73.3%) studies employed Diagnostic and Statistical Manual of Mental Disorders (DSM) as depression diagnostic criteria. The number of patients allocated to each group ranged between five and 60. The settings for recruited population were inpatients (66.7%), outpatient (20.0%) and community-based (13.3%). One trial only enrolled patients diagnosed with major depression, five trials recruited patients with both major and minor depression, and the rest nine trials did not specify. The median treatment duration was 8 weeks (range 4–16), while the follow-up duration ranged from 4 weeks to 18 months. Nearly half of trials were multicenter site studies (40.0%). Five (33%) trials recruited patients from North America, six (40%) from Europe, three (20%) from China and the rest one (7%) from Australia. The patient characteristics of the NMA are summarized in Table 2 of Appendix 4. Across trials, patient mean age ranged from 51.1 to 76.0 years and approximately half of the sample population were male (52.9%). The mean baseline Hamilton Depression Scale (HAMD) score ranged from 13.9 to 32.8. More detailed description of the studies and treatments is provided in Appendix 4.

**Table 1:** Study characteristic

Study	Location	Participants (*N*)	Intervention/ Control (*N*)	Drop-out Rate (%)	Treatment Duration	Follow-up	Setting	Center	Depression Diagnostic Criteria	Population
Lipsey 1984	US	39	Nortriptyline 17 Placebo 22	35.3 31.8	6 weeks	6 weeks	mixed	multi-center	DSM III	PP
Andersen 1994	Denmark	66	Citalopram 33; Placebo 33	21.2 6.06	6 weeks	16 weeks	mixed	multi-center	DSM III	ITT, PP
Torrecillas 1995	Belgium	48	Fluoxetine 26 Nortriptyline 11 Placebo 11	3.8 9.1 9.1	6 weeks	6 weeks	inpatient	single center	RDC	PP
Miyai 1998	US	24	Desipramine 13 Trazodone 6 Fluoxetine 5	38.5 0 20	4 weeks	4 weeks	inpatient	single center	DSM III	PP
Robinson 2000	US	56	Fluoxetine 23 Nortriptyline 16 Placebo 17	39.1 18.8 23.5	12 weeks	12 weeks	inpatient	multi-center	DSM-IV	ITT,PP
Kimura 2000	US	47	Nortriptyline 21 Placebo 26	14.3 0	6 or 12 weeks	12 weeks	inpatient	multi-center	DSM-IV	PP
Fruehwald 2003	Austria	54	Fluoxetine 28; Placebo 26	7.14 7.7	12 weeks	18 months	inpatient	multi-center	NR	PP
Kimura 2003	US	27	Nortrityline 13 Placebo 14	7.7 0	6 or 12 weeks	12 weeks	inpatient	multi-center	DSM-IV	ITT
Rampello 2003	Italy	74	Citalopram 37 Reboxetine 37	8.1 8.1	16 weeks	16 weeks	outpatient	community-based	DSM-IV	PP
Rampello 2004	Italy	31	Reboxetine 16 Placebo 15	0	16 weeks	16 weeks	outpatient	community-based	DSM-IV	ITT
Huang 2005	China	60	Fluoxetine 30 Clomipramine 30	0	12 weeks	12 weeks	inpatient	single center	CCMD	ITT
Ye 2006	China	90	Paroxetine 30 Imipramine 30 Placebo 30	3.3 1 1	12 weeks	12 weeks	inpatient	single center	NR	PP
Li 2008	China	150	TCM 60 Fluoxetine 60 Placebo 30	0 3.3 6.7	8 weeks	8 weeks	inpatient	single center	NR	ITT
Cravello 2009	Italy	50	Fluoxetine 25 Venlafaxine 25	0	8 weeks	8 weeks	inpatient	single center	DSM-IV	ITT
Karaiskos 2012	Greece	60	Duloxetine 20 Citalopram 20 Sertraline 20	0	3 months	3 months	outpatient	single center	DSM-IV	ITT

DSM = Diagnostic and Statistical Manual of Mental Disorders; DSM-IV-TR = Diagnostic and Statistical Manual of Mental Disorders fourth edition, text revision; RDC = Research Diagnostic Criteria; CCMD = Chinese Classification of Mental Disorder; TCM = traditional Chinese medicine; CES-D = center of epidemiological survey depression scale; NR = not reported; PP = per protocol; ITT = intention to treat.

**Table 2:** Summary effect size of pairwise and network meta-analysis

Comparisons	No. of trials	Pairwise meta-analysis mean difference/odds ratios (95% CI)	*P*-value	Heterogeneity I^2^	Network meta-analysis mean difference/odds ratios (95% CrI)	Quality of evidence	Downgraded reason
**Primary Main Outcomes (Effects after completion of treatments)**							
**Paroxetine VS Placebo**	1	NA	NA	NA	**13.49 (4.36,22.52)**	⊕⊕OO low	imprecision and indirectness
**Imipramine VS Placebo**	1	NA	NA	NA	**11.37 (2.12,20.33)**	⊕⊕OO low	imprecision and indirectness
**Reboxetine VS Placebo**	1	NA	NA	NA	**9.57 (2.37,16.58)**	⊕⊕OO low	imprecision and indirectness
**Nortriptyline VS Placebo**	5	**7.67 (2.65, 12.69)**	0.00	99.00	**7.61 (3.51,11.65)**	⊕⊕⊕O moderate	heterogeneity
**Citalopram VS Placebo**	1	NA	NA	NA	**7.49 (0.19,14.50)**	⊕⊕OO low	imprecision and indirectness
**Fluoxetine VS Placebo**	4	5.31 (–1.99,12.62)	0.00	96.00	**4.91 (0.26,9.32)**	⊕⊕OO low	inconsistency and heterogeneity
**Short-term Outcomes (Efficacy of 4-week duration)**							
**Duloxetine VS Placebo**	1	NA	NA	NA	**13.22 (2.52,23.48)**	⊕⊕OO low	imprecision and indirectness
**Reboxetine VS Placebo**	1	NA	NA	NA	**9.25 (2.24,16.31)**	⊕⊕OO low	imprecision and indirectness
**Trazodone VS Placebo**	1	NA	NA	NA	**9.29 (0.82,17.48)**	⊕⊕OO low	imprecision and indirectness
**Fluoxetine VS Placebo**	4	**4.89 (0.09, 9.69)**	0.00	93.00	**6.18 (2.07,10.22)**	⊕⊕⊕O moderate	heterogeneity
**Nortriptyline VS Placebo**	4	**4.44 (0.29, 8.60)**	0.00	99.00	**4.28 (0.60,7.85)**	⊕⊕⊕O moderate	heterogeneity
**Duloxetine VS Citalopram**	1	NA	NA	NA	**8.20 (0.36,15.85)**	⊕	imprecision and indirectness
**Duloxetine VS Sertraline**	1	NA	NA	NA	**9.05 (0.75,16.96)**	very low	risk of bias and imprecision and indirectness
**Medium-term Outcomes (Efficacy of 8-week duration)**							
**Duloxetine VS Placebo**	1	NA	NA	NA	**12.52 (1.42,23.30)**	⊕⊕OO low	imprecision and indirectness
**Paroxetine VS Placebo**	1	NA	NA	NA	**12.12 (3.10,20.10)**	⊕⊕OO low	imprecision and indirectness
**Imipramine VS Placebo**	1	NA	NA	NA	**10.93 (2.04,19.38)**	⊕⊕OO low	imprecision and indirectness
**Reboxetine VS Placebo**	1	NA	NA	NA	**8.71 (2.13,15.24)**	⊕⊕OO low	imprecision and indirectness
**Nortriptyline VS Placebo**	5	**7.09 (2.04,12.13)**	0.00	99.00	**6.98 (2.79,10.56)**	⊕⊕⊕O moderate	imprecision
**Citalopram VS Placebo**	1	NA	NA	NA	**6.92 (0.18,13.61)**	⊕⊕OO low	imprecision and indirectness
**Fluoxetine VS Placebo**	4	**6.12 (0.50,12.73)**	0.00	96.00	**5.99 (1.48,10.24)**	⊕⊕⊕O moderate	imprecision
**Secondary Outcomes of Response Rate**							
**Nortriptyline VS Placebo**	3	**4.90 (2.02,11.87)**	0.70	0.00	**8.33 (2.44,25.00)**	⊕⊕⊕O moderate	imprecision
**Nortriptyline VS Clomipramine**	1	NA	NA	NA	**16.67 (1.12,100.00)**	⊕⊕OO low	imprecision and indirectness
**Nortriptyline VS Fluoxetine**	1	NA	NA	NA	**16.67 (3.00,100.00)**	very low	risk of bias and imprecision and indirectness
**Nortriptyline VS TCM**	1	NA	NA	NA	**20.00 (2.00,100.00)**	⊕⊕OO low	imprecision and indirectness
**Paroxetine VS Fluoxetine**	1	NA	NA	NA	**12.50 (1.35,100.00)**	⊕⊕OO low	imprecision and indirectness
**Imipramine VS Fluoxetine**	1	NA	NA	NA	**11.11 (1.18,100.00)**	⊕⊕OO low	imprecision and indirectness

TCM, Traditional Chinese Medicine; NA, not applicable; 95% CI, 95% Confidence Intervals; 95% CrI, 95% Credible Intervals. Results are expressed as odds ratios with 95% CI or 95% CrI for dichotomous variables (response). While the mean difference with 95% CI or 95% CrI was used for continuous outcomes (maximal voiding volume, voiding frequency, incontinence episodes and average voiding volume). Significant results are in bold.

### Meta-analysis results

Data analysis on the efficacy outcomes are summarized in Figure 3. Table 2 shows the available direct comparisons and network of trials for efficacy.

**Figure 2:**
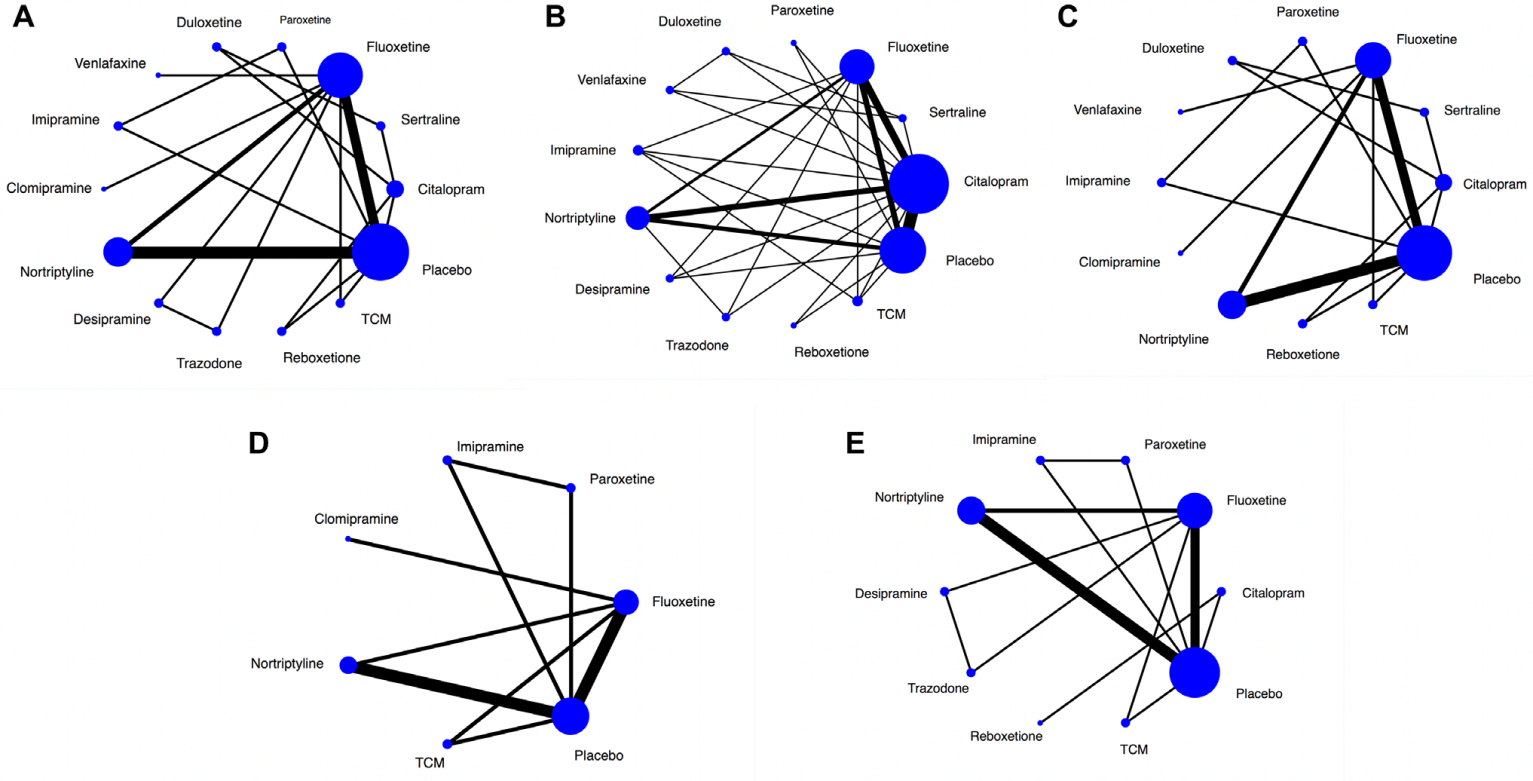
Network of eligible comparisons The width of the lines is proportional to the number of trials comparing every pair of treatments, and the size of every circle is proportional to the number of randomly assigned participants (sample size). TCM = Traditional Chinese Medicine. (**A**) Network of eligible comparisons for efficacy after treatment completion. (**B**) Network of eligible comparisons for efficacy of four-week duration. (**C**) Network of eligible comparisons for efficacy of eight-week duration. (**D**) Network of eligible comparisons for response rate. (**E**) Network of eligible comparisons for tolerability.

**Figure 3:**
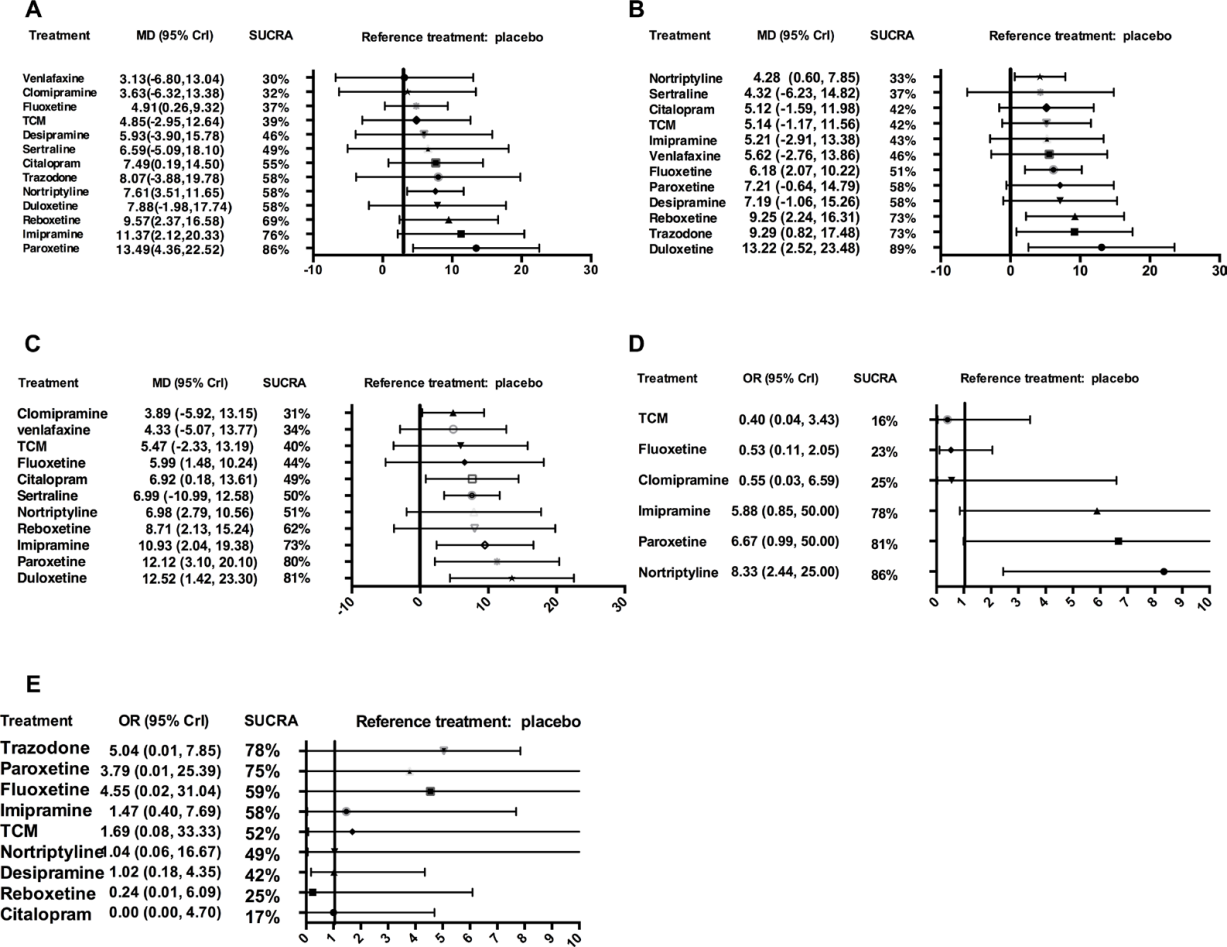
Forest plot of network meta-analysis results Treatments are reported in order of efficacy ranking according to SUCRAs. All treatments are compared to placebo. (**A**) Summary mean difference and credible intervals from network meta-analysis of HAMD score change at the end of treatment; (**B**) Summary mean difference and credible intervals from network meta-analysis of HAMD score change at four-week; (**C**) Summary mean difference and credible intervals from network meta-analysis of HAMD score change at eight-week; (**D**) Summary odds ratio and credible intervals from network meta-analysis of response rate; (**E**) Summary odds ratio and credible intervals from network meta-analysis of adverse events; MD = mean difference, OR = odds ratio, CrI = credible intervals, SUCRA = surface under the cumulative ranking curve, TCM = Traditional Chinese Medicine.

### Pairwise meta-analysis

Forest plot of direct comparisons for efficacy and tolerability are seen in Appendix 5. Results of direct pairwise meta-analysis are summarized in Appendix 6. In terms of HAMD score change at the end of treatment, paroxetine, imipramine, nortriptyline, citalopram and Chinese Traditional Medicine (TCM) were significantly better than control group. Considering short-term (4-week effects) and medium-term (8-week effects) outcomes, paroxetine, nortriptyline, citalopram, TCM, fluoxetine was associated with a pronounced HAMD score improvement compared with control group. Duloxetine was significantly better than citalopram and sertraline. However, paroxetine and imipramine only showed priority to control group in short-term outcome, because they were not included in the medium-term outcome.

### Network meta-analysis efficacy outcomes

#### Primary outcomes

For the main analysis of our primary outcomes (effects after completion of treatments), 13 treatment arms formed our analysis. (Figure 2A) Fluoxetine, nortriptyline and placebo are the three most frequent comparators across the studies. Of these, nine trials were two-arm and six were three-arm. All antidepressant drugs were directly compared with at least one other active drug, and eight drugs had at least one placebo-controlled trial. The NMA showed that five antidepressant agents (paroxetine, imipramine, reboxetine, nortriptyline, citalopram and fluoxetine) had a significant advantage over placebo (mean difference [MD] 13.49, 95% credible interval [Crl] 4.36 to 22.52, cumulative ranking curve [SUCRA] = 86%; MD 11.37, 95% Crl 2.12 to 20.33, SUCRA = 76%; MD 9.57, 95% Crl 2.37 to 16.58, SUCRA = 69%; MD 7.61, 95% Crl 3.51 to 11.65, SUCRA = 58%; MD 7.49, 95% Crl 0.19 to 14.50, SUCRA = 55%; MD 4.91, 95% Crl 0.26 to 9.32, SUCRA = 37%). However, there were no significant differences in any head-to-head comparisons (Figure 3A and Table 1 of Appendix 6).

Twelve trials reported short-term outcomes (efficacy of 4-week duration), involving 12 drugs (except for clomipramine) (Figure 2B). Different from the main outcomes, an unexpected finding was that duloxetine showed superior advantages. In the NMA, duloxetine ranked the best for overall change in HAMD score (SUCRA = 89%), and was found to be superior to the placebo (MD 13.22, 95% Crl 2.52 to 23.48), followed by reboxetine, trazodone, fluoxetine and nortriptyline (MD 9.25, 95% Crl 2.24 to 16.31; MD 9.29, 95% Crl 0.82 to 17.48; MD 6.18, 95% Crl 2.07 to 10.22; MD 4.28, 95% Crl 0.60 to 7.85). Moreover, duloxetine was superior to citalopram and sertraline (MD 8.20, 95% Crl 0.36 to 15.85; MD 9.05, 95% Crl 0.75 to16.96) (Figure 3B and Table 2 of Appendix 6).

The outcomes of medium-term (efficacy of 8-week duration) including 13 drugs broadly agree with 4-week efficacy results (Figure 3C), except for paroxetine (MD 12.12, 95% Crl 3.10 to 20.10) and imipramine (MD 10.93, 95% Crl 2.04 to 19.38) which both performed better in the 8-week efficacy results. More interestingly, duloxetine ranked the best (SUCRA = 81%) and was significantly superior to SSRIs (citalopram and sertraline) within 4-week period but not at the endpoint of 8-week or the whole study (3-month) (Figure 3C and Table 3 of Appendix 6).

### Secondary outcomes

NMA for secondary outcomes of response rate did not differ significantly from the findings of our primary outcomes (Figure 2D and Table 4 of Appendix 6). The only exception was nortriptyline, which was more efficacious in the secondary analysis, because it ranked the best and was much better than the placebo (odds rate [OR] 8.33, 95% CrI 2.44 to 25.00; SUCRA = 86%) and three other agents (clomipramine, fluoxetine and TCM). Besides, paroxetine and imipramine were associated with significantly better response rates than reboxetine (Figure 3D and Table 5 of Appendix 6).

### Network meta-analysis of tolerability outcomes

Four trials were excluded from this analysis for reporting zero events across all treatment arms (Figure 3E). In this NMA, overall tolerability outcome was represented by data on discontinuation of the whole trial protocols. We did not observe significant differences between any head-to-head comparisons, or any antidepressant-treated group and placebo group. However, according to the ranking, there is a trend that trazodone, paroxetine and fluoxetine were associated with a relatively better tolerability, while citalopram, reboxetine, desipramine and nortriptyline were less tolerable. Moreover, the 95% credible intervals were relatively wide. Common adverse events in these treatment group included CNS symptoms (e.g., headache, sedation, tremor, fatigue, insomnia), gastrointestinal symptoms, and vascular events (e.g., dizziness, palpitation) (Table 5 of Appendix 6).

### Sensitivity analysis

With respect to the trials involved patients with HAMD score higher than 20 only, NMA was repeated using change of HAMD score at the end of treatment. We observed a significant superiority of paroxetine, imipramine, reboxetine, nortriptyline, citalopram and fluoxetine over control (Appendix 7). The results were similar to those of the full analysis, but the power was compromised because of the small sample size.

### Quality assessment and quality of the evidence

The risk of bias for random sequence generation in eight trials was high or unclear; concealment of treatment allocation in eight trials; masking of participants, masking of investigators, or both in four trials; incompleteness of outcome reporting in two trials and selective reporting of outcomes in two trials. None of the studies accepted financial funding from commercial bodies and source of funding was unclear in ten trials. (Appendix 8) We did not find any evidence of small study effects based on funnel plot asymmetry, though the number of studies included in each comparison was small. Generally, there was no obvious risk of bias, indirectness, inconsistency, or publication bias for any of the direct comparisons. In several comparisons, there was serious imprecision in summary estimate because the 95% credible interval crossed unity. According to GRADE, the quality of evidence for most results were low or very low. Nevertheless, we had moderate confidence in estimates supporting the use of nortriptyline for primary efficacy outcomes and secondary outcome of response rate, and moderate confidence in estimates supporting the use of fluoxetine for 4-week efficacy and 8-week efficacy outcomes. Conceptually, there was no significant intransitivity. The GRADE quality of evidence supporting the use of each treatment for primary outcome was summarized in the Appendix 6.

### Network consistency

There was no inconsistency in the NMA estimates when we used the node-splitting approach and no significant differences between direct and indirect estimates in closed loops that allowed assessment of network coherence. The total residual deviance for overall change in HAMD score at endpoint (89.1, df = 91), short-term (74.3, df = 78), and medium term (64.9, df = 66) implied a good model fit. Convergence of chains was verified visually by looking at trace plots and inspecting the Brooks-Gelman-Rubin diagnostic statistics with values around 1.

## DISCUSSION

This NMA confers us a relatively complete profile of the efficacy and tolerability of antidepressant agents for patients with PSD. From this NMA, the pooled analysis revealed that paroxetine, imipramine, reboxetine, nortriptyline and citalopram showed statistically significant treatment efficacy when compared with placebo in PSD patients in reducing HAMD score at the end of treatments. Intriguingly, we observed a little difference among analysis of 4-week, 8-week efficacy and the results after completion of treatments. Further secondary analysis of response rates also showed relatively consistent positive effects of antidepressants on treating PSD. We did not observe significant difference among the drugs in tolerability analysis.

Paroxetine ranked highest in our primary main outcome, probably because it is one of the first two potent inhibitors of serotonin re-uptake among six SSRIs [25], works by selectively inhibiting the neuronal presynaptic reuptake of serotonin to facilitate serotoninergic neurotransmission [26, 27]. In consistent with other studies broadly [28–30], our NMA demonstrated that paroxetine was significantly superior to placebo and comparable to other drugs like imipramine, clomipramine and so on in relieving depressive symptoms. However, this evidence could be less convincing for the relatively low quality and small sample size. Apart from the effects on depression, evidence also showed that paroxetine effectively improved the cognitive and functional performance of patients with PSD, as well as their quality of life [31]. However, a large study (*n* = 13 741) conducted by the UK Drug Safety Research Unit reported that male sexual dysfunction was more common with paroxetine than fluoxetine [32], which indicated that paroxetine could be a particular disadvantage for stroke survivors with compromised sexual function.

An intriguing finding is that duloxetine, as a SNRI antidepressant, was proved to be clearly superior to SSRIs (citalopram and sertraline) with a faster and significantly more reduction of depressive symptoms especially in the first month of treatment [33], which may be attributed to its double selective reuptake-inhibiting effect on serotonergic and noradrenergic neurotransmission [34, 35]. While SSRIs are known to inhibit only serotonergic receptors, SNRIs act on noradrenergic receptors as well. However, the significant difference disappeared at the endpoint of the whole study, which may be attributed to the gradually increased antidepressant action of SSRIs during the longer duration of treatment. In other words, the effectiveness of SSRIs increased, while the effectiveness of duloxetine disappeared with longer term treatment. Thus, for aged patients with underlying diseases, duloxetine may be a better choice in order to get an early improvement. Besides, a rapid onset of antidepressant action may also be useful for decreasing the risk of suicide and depressive relapse, and shorten days of hospitalization [36].

As far as response rate, nortriptyline and paroxetine ranked high in the SUCRA analysis, and was both numerically superior to fluoxetine. Particularly, if no response to antidepressants has been shown for 6 weeks, there would be no benefit to continue the medication, which means medication should be changed [37]. Therefore, based on the results of our NMA, it may be appropriate to try paroxetine for patients who did not show response to previous antidepressant, for paroxetine seems to have excellent potential in treating post-stroke depression.

With regard to tolerability, no statistically significant differences were detected. Insufficient data for assessing adverse effects may be one of the reasons underlying this result. Larger sample sizes and more specific assessment of adverse events might be helpful to reveal the potential harmful effects of antidepressants. Another possible explanation is the drop-out rate may not reflect the real adverse events rate. Nonetheless, there is still a trend that antidepressants were less well tolerated for some adverse effects. This suggests that one should be extremely cautious when prescribing antidepressants for stroke survivals with PSD because they are particularly vulnerable to the adverse effects. Many stroke patients with underlying cardiovascular problems are on polytherapy, which may lead to drug interactions. For example, SSRIs may increase the effects of anticoagulant agents, leading to increased risk of hemorrhage when administered with analgesic agents (NSAIDs or acetylsalicylic acid) [38], probably owing to their direct action on platelet function mediated through inhibiting platelet serotonin uptake [39] or inhibition of cytochrome P450 enzymes in the liver [40]. Thereby, anti-coagulation therapy requires particularly careful observation when SSRIs are prescribed. TCAs may increase or reduce anticoagulant effects of anticoagulant agents, and increase risk of ventricular arrhythmias when used with antibiotic (e.g. moxifloxacin) or antiarrhythmic agents [38]. Therefore, physicians should review the adverse effect profiles of different drug classes to choose an agent meeting the clinical demands of the exact patient.

Our meta-analysis has several weaknesses. First, risk of bias and methodological shortages within individual studies, and the relatively small sample size of some trials, which may restrict our findings for clinical application. Particularly, the existing variations in the characteristics of recruited participants (e.g., age, time after stroke onset and different doses of antidepressants) may also introduce potential bias. Hence, although our NMA currently represents the best available evidence, it may not be necessarily the best possible evidence. Thus, our findings are not decisive and more high-quality RCTs with appropriate duration in patients with PSD were needed. Second, we only retrieved trials used HAMD scale for the purpose of performing statistical analysis, which may to some extent introduce selective bias. However, HAMD has been taken as a gold standardized scale of the severity of depression for over 50 years, and it was ideally suited to measure the effects of drug treatments [41, 42]. Besides, this objective clinician scoring instrument, mainly based on clinician observations, not only gained popularity among physicians because it is simple to use, but also is well accepted by patients for making them feel that the clinicians are quite familiar with their conditions, including psychological and somatic symptoms [43]. Third, several studies excluded participants with some complications (e.g., aphasia, cognitive impairment, or history of psychiatric illness) and such strict exclusion criteria probably weaken the external validity, for a large proportion of stroke survivors would be excluded and the rest were presumably not representative of patients with PSD requiring management in the “real word” [44, 45]. Furthermore, the accuracy of the results may be affected by missing data in some non-English publications and unpublished trials with negative results. Finally, the wide time range of the included publications (1984–2012) might introduce heterogeneity.

The guidelines of the American College of Physicians recommended that antidepressant therapy should last for at least 4 months to prevent recurrence. Therefore, further research should manage to conduct larger multicenter RCTs with long-term follow-up, which is necessary to identify effective and safe antidepressants for PSD, as well as the optimal duration and dose to maximize therapeutic benefits. In addition, future studies should focus on relapse prevention as well as long-term maintenance, and carry out subgroup analysis based on diverse length of time between beginning of depression and onset stroke, for depression occurring in the early phase of stroke may differ from the one appearing several months post stroke.

## MATERIALS AND METHODS

This systematic review is reported according to the Preferred Reporting Items for PRISMA statement extension for NMA (Appendix 1) [46] and was performed according to a priori established protocol registered with PROSPERO (CRD42016049049) [47].

Details of the methods have been depicted in our priori published protocol. We also reviewed trials on non-pharmacological interventions (e.g. psychotherapy, repetitive transcranial magnetic stimulation). Because trials of pharmacological interventions differ from non-pharmacological interventions in terms of patients, recruitment strategies, control interventions and outcomes, these pharmacological trials are decided to be analyzed separately.

### Search strategy and study selection

To compare the safety and efficacy of different drugs for PSD treatment, we captured RCTs published in English and up to March 1, 2017, and compiled from the following databases: PubMed, Embase and the Cochrane Library Central Register of Controlled Trials (CENTRAL). We manually screened references from published systematic reviews in the discipline and retrieved trials. A modified search algorithm for each database was adapted (Appendix 2).

We captured RCTs that attempted to evaluate antidepressants at licensed doses of these medications with other drugs or placebo in the management of patients with PSD. Patients had to be diagnosed with stroke (ischemic or hemorrhagic), and depression according to specific criteria (e.g., DSM or depression scales (e.g., HAMD). Only trials using the HAMD scale for assessing the depression degree of patients were included, with reported results of mean score change from baseline to post-treatment, or the proportion of patients responding to treatment (defined as a reduction of more than 50% in HAMD score), or study discontinuation for any reason. Three independent investigators (LHD, SQ and YY) initially screened all the trials. Disagreements were resolved by discussion.

### Comparison and outcomes

For efficacy analysis, the primary outcome was the mean change in HAMD between baseline and endpoint. The secondary outcome was defined as the proportion of patients who responded to treatment (showing a decrease of ≥ 50% measured by HAMD score).

For our main efficacy analysis, when a trial reported multiple data with different endpoints, we collected data after completion of the whole treatments (e.g., the treatment duration was eight-week while data were provided weekly, we gave priority to the data of eight-week). Moreover, we used the measurement points closest to four-week and eight-week respectively, to conduct analysis of short- and medium-term effects. For tolerability analysis, the outcome was presented by the discontinuation (dropout rate) for any reason.

### Data extraction and statistical analysis

Two reviewers (LHD, SQ) extracted the relevant information from the obtained publications with a pre-established data extraction sheet. An approximation of the mean was used to evaluate the outcomes, where data were merely available in graphic format. The highest standard deviations in the HAMD scores from the other trials were recruited when data were presented without standard deviations [48]. For tolerability analysis, we excluded studies in which zero drop-out events were reported across all treatment arms.

For outcomes for which two or more trials comparing two interventions were available, we firstly conducted a conventional pairwise meta-analysis applying random-effects model option [49]. To combine direct and indirect evidence, we conducted fixed-effects Bayesian NMA employing Markov chain Monte Carlo methods in WinBUGS version 1.4.3. A Bayesian fixed-effect framework was considered appropriate due to the limited number of trials concerning each edge in the network [50, 51]. The results of NMA with effect sizes (MD or OR) and their credible intervals (CrI) were summarized. We evaluated the relative ranking probability of each strategy and obtained the hierarchy of competing interventions using rankograms and surface under the SUCRA [52]. To check for inconsistency, we employed the node-splitting method [53]. To check for the assumption of consistency in the entire network, the design-by-treatment model was constructed [51]. Finally, sensitive analysis was performed to value the robustness of the findings.

### Risk of bias and quality of evidence

The validity of the meta-analysis was assessed by qualitative appraisal of study designs and methods. Two independent assessors (LHD, SQ) assessed risk of bias using the Cochrane Collaboration Handbook [48], focusing on selection bias, information bias, and bias in the analysis. We used the funnel plot to detect publication bias, only when at least ten studies were available [48]. The GRADE methodology was performed to rate the quality of evidence. In this approach, direct evidence from RCTs starts at high quality and can be downgraded based on risk of bias, imprecision, indirectness, inconsistency (or heterogeneity) and publication bias to levels of moderate, low and relatively low quality [54].

## CONCLUSIONS

Using randomized trial data and a novel evidence synthesis approach, we evaluated the relative efficacy and tolerability of the available pharmacological interventions for PSD. Our results suggest that paroxetine, imipramine, reboxetine, nortriptyline, citalopram and fluoxetine are associated with significant HAMD score reduction compared with placebo at the end of treatment. In order to get a quicker relief of depression, duloxetine may be useful for its rapid onset of antidepressant action. The tolerability was comparable among all the antidepressants. More high-quality RCTs are needed in this fields. Future studies should focus on long-term effectiveness and tolerability of antidepressants, and investigate the optimal timing and thresholds for treatments associated with the highest response and remission rate.

## SUPPLEMENTARY MATERIALS FIGURES AND TABLES




