# Supplementary material for: Efficacy and tolerability of pharmacotherapy for post-stroke depression: a network meta-analysis
Source: Oncotarget. 2018 Jan 3;9(34):23718–28. doi: 10.18632/oncotarget.23891 (PMC5955092; doi:10.18632/oncotarget.23891)
Supplement: Supplementary file 1 [file oncotarget-09-23718-s001.pdf]

Efficacy and tolerability of pharmacotherapy for post-stroke depression: a network meta-analysis

SUPPLEMENTARY MATERIALS

Supplementary Appendix: Appendices 1-8. See Supplementary_Appendix
