# Supplementary material for: Efficacy and tolerability of pharmacotherapy for post-stroke depression: a network meta-analysis
Source: Oncotarget. 2018 Jan 3;9(34):23718–28. doi: 10.18632/oncotarget.23891 (PMC5955092; doi:10.18632/oncotarget.23891)
Supplement: Supplementary file 2 [file oncotarget-09-23718-s002.docx]

**Additional file 1: Appendices 1-8**

**Abbreviations**

**Appendix 1: PRISMA network meta-analysis checklist**

**Appendix 2: Search algorithms**

**Appendix 3: References for included trials**

**Appendix 4: Description of included studies, outcomes**

**Appendix 5: Forest plot of direct pairwise meta-analysis for efficacy and safety**

**Appendix 6: Results and quality of evidence of network meta-analysis**

**Appendix 7: Statistically significant results of network meta-analysis for sensitive analysis.**

**Appendix 8: Risk of bias assessments within studies**

**Abbreviations**

PSD= post-stroke depression

HAMD= Hamilton Depression Rating Scale(or HRSD)；

DSM= Diagnostic and Statistical Manual of Mental Disorders

RCT= traditional Chinese medicine

SSRI= selective serotonin reuptake inhibitor

TCA= tricyclic antidepressant

SNRI= serotonin–norepinephrine reuptake inhibitors

NRI= norepinephrine reuptake inhibitor

TCM= traditional Chinese medicine

MMSE= Mini-mental State examination;

NR=not reported

SD= standard deviation

ITT = intention-to-treat

PP= per protocol

BDI=Beck Depression Inventory

CT=computed tomography

MRI=magnetic resonance imaging

SAH=subarachnoid hemorrhage

TIA=transient ischemic attack

**Appendix 2: Search algorithms**

| Medline | |
| --- | --- |
| #1 | “randomized controlled trial” [Publication Type] |
| #2 | “controlled clinical trial” [Publication Type] |
| #3 | “randomized” [Title/Abstract] |
| #4 | “randomly” [Title/Abstract] |
| #5 | “trial” [Title] |
| #6 | “Randomized Controlled Trial as Topic” [MeSH] |
| #7 | (#1) OR (#2) OR (#3) OR (#4) OR (#5) OR (#6) |
| #8 | “PSD” [Title/Abstract] |
| #9 | “post-stroke depression” [Title/Abstract] |
| #10 | “post-stroke depressive”[Title/Abstract] |
| #11 | “depression after stroke”[Title/Abstract] |
| #12 | “depression in stroke patients”[Title/Abstract] |
| #13 | “depression after cerebral apoplexy”[Title/Abstract] |
| #14 | “depression after cerebrovascular accident”[Title/Abstract] |
| #15 | “depression after cerebrovascular disease”[Title/Abstract] |
| #16 | (#8) OR (#9) OR (#10) OR (#11) OR (#12) OR (#13) OR (#14) OR (#15) |
| #17 | “Therapeutics”[Mesh] OR “Antidepressive Agents” [Mesh] |
| #18 | “Serotonin Uptake Inhibitors” [Mesh] OR “Fluoxetine” [Mesh] OR “Sertraline” [Mesh] OR “Paroxetine” [Mesh] OR “Citalopram” [Mesh] OR “Fluvoxamine” [Mesh] OR “Escitalopram” [Title/Abstract] |
| #19 | “Antidepressive Agents, Tricyclic” [Mesh] OR “Nortriptyline” [Mesh] OR “Imipramine” [Mesh] OR “Clomipramine” [Mesh] OR “Amitriptyline” [Mesh] |
| #20 | “Serotonin norepinephrine reuptake inhibitor” [Title/Abstract] OR “SNRI” [Title/Abstract] OR “Venlafaxine” [Mesh] OR “Duloxetine” [Mesh] OR “NRI” [Title/Abstract] OR “reboxetine” [Title/Abstract] |
| #21 | “Monoamine Oxidase Inhibitors” [Mesh] OR “Methylphenidate” [Mesh] OR “aniracetam” [Title/Abstract] OR “psychostimulant” [Title/Abstract] |
| #22 | “Drugs, Chinese Herbal” [Mesh] |
| #23 | (#17) OR (#18) OR (#19) OR (#20) OR (#21) OR (#22) |
| #24 | (#7) AND (#16) AND (#23) |
| 192 | |

| Embase | |
| --- | --- |
| #1 | ‘randomized controlled trial’/exp: ti,ab,kw |
| #2 | ‘randomized controlled trial (topic)’/exp: ti,ab,kw |
| #3 | random *: ti,ab,kw |
| #4 | #1 OR #2 OR #3 |
| #5 | ‘PSD’/exp: ti,ab,kw |
| #6 | ‘post-stroke depression’/exp: ti,ab,kw: ti,ab,kw |
| #7 | ‘post-stroke depressive’/exp: ti,ab,kw |
| #8 | ‘depression after cerebral apoplexy’/exp: ti,ab,kw |
| #9 | ‘depression after cerebrovascular accident’/exp: ti,ab,kw |
| #10 | ‘depression after cerebrovascular disease’/exp: ti,ab,kw |
| #11 | ‘depression in stroke patients’/exp: ti,ab,kw |
| #12 | ‘depression after stroke’ /exp: ti,ab,kw |
| #13 | #5 OR #6 OR #7 OR #8 OR #9 OR #10 OR #11 OR #12 OR |
| #14 | ‘therapeutics’ /exp: ti,ab,kw OR ‘antidepressive Agents’ /exp: ti,ab,kw |
| #15 | ‘tricyclic’ /exp: ti,ab,kw |
| #16 | ‘serotonin uptake inhibitors’ /exp: ti,ab,kw |
| #17 | ‘serotonin norepinephrine reuptake inhibitor’ /exp: ti,ab,kw |
| #18 | ‘monoamine oxidase inhibitors’ /exp: ti,ab,kw |
| #19 | ‘fluoxetine’ /exp: ti,ab,kw OR ‘sertraline’ /exp: ti,ab,kw OR ‘paroxetine’ /exp: ti,ab,kw OR ‘citalopram’ /exp: ti,ab,kw |
| #20 | ‘Chinese Herbal’ /exp: ti,ab,kw |
| #21 | (#14) OR (#15) OR (#16) OR (#17) OR (#18) OR (#19) OR (#20) |
| #22 | (#4) AND (#13) AND (#21) |
| 247 | |

| Cochrane Library Central | |
| --- | --- |
| #1 | PSD: ti, ab, kw OR post- stroke depression: ti, ab, kw in Trials (Word variations have been searched) |
| #2 | depression after cerebrovascular disease: ti, ab, kw OR depression after cerebral apoplexy: ti, ab, kw in Trials (Word variations have been searched) |
| #3 | depression after stroke: ti, ab, kw in Trials (Word variations have been searched) |
| #4 | depression in stroke patients : ti, ab, kw in Trials (Word variations have been searched) |
| #5 | (#1) OR (#2) OR (#3) OR (#4) |
| #6 | therapeutics: ti, ab, kw OR antidepressive: ti, ab, kw OR serotonin reuptake inhibitor: ti, ab, kw OR tricyclic: ti, ab, kw OR monoamine oxidase inhibitor: ti, ab, kw in Trials (Word variations have been searched) |
| #7 | fluoxetine: ti, ab, kw OR sertraline: ti, ab, kw OR paroxetine: ti, ab, kw OR citalopram: ti, ab, kw OR reboxetine: ti, ab, kw in Trials (Word variations have been searched) |
| #8 | trazodone: ti, ab, kw OR nortriptyline: ti, ab, kw OR escitalopram: ti, ab, kw OR psychostimulant: ti, ab, kw OR Chinese herbal medicine: ti, ab, kw in Trials (Word variations have been searched) |
| #9 | (#6) OR (#7) OR (#8) |
| #10 | (#5) AND (#9) |
| 425 | |

**Appendix 3: References for included trials**

1. Lipsey JR, Robinson RG, Pearlson GD, Rao K, Price TR. Nortriptyline treatment of post-stroke depression: a double-blind study. Lancet. 1984; l(8372):297-300.
2. Andersen G, Vestergaard K, Lauritzen L, General F. Original contributions effective treatment of post-stroke depression with the selective serotonin reuptake inhibitor citalopram. Stroke. 1994;25(6):1099-1104.
3. Gonzalez-torrecillas JL, Mendlewicz J, Lob A. Effects of early treatment of post-stroke depression on neuropsychological rehabilitation. Int Psychogeriatr. 1995;7(4):547-560
4. Miyai I, Reding MJ. Effects of antidepressants on functional recovery following stroke: a double-blind study. J Neuro Rehab. 1998;12(1):5–13.
5. Robinson RG, Schultz SK, Castillo C, Kopel T, Kosier JT, Newman RM, et al. Nortriptyline versus fluoxetine in the treatment of depression and in short-term recovery after stroke. Am J Psychiatry. 2000;157(3):351–359.
6. Kimura M, Robinson RG, Kosier JT. Treatment of cognitive impairment after post-stroke depression: A double-blind treatment trial. Stroke.2000;31(31):1482–1486.
7. Fruehwald S, Gatterbauer E, Rehak P, Baumhackl U. Early fluoxetine treatment of post-stroke depression: A three-month double-blind placebo-controlled study with an open-label long-term follow up. J Neuro. 2003;250(3):347–351.
8. Kimura M, Tateno A, Robert G, Robinson M. Treatment of post-stroke generalized anxiety disorder comorbid with post-stroke depression merged analysis of nortriptyline trials. Am J Geriatr Psychiatry;2003;11(11):320–327
9. Rampello L, Chiechio S, Nicoletti G, Alvano A, Vecchio I, Raffaele R. Prediction of the response to citalopram and reboxetine in post-stroke depressed patients. Psychopharmacology. 2004;173(1-2):73–78.
10. Rampello L, Alvano A, Chiechio S. An evaluation of efficacy and safety of reboxetine in elderly patients affected by “retarded” post-stroke depression: A random, placebo-controlled study. Arch Gerontol Geriatr. 2004;40(3):275–285.
11. Huang L. Comparison of therapeutic effects and side effects between fluoxetine hydrochloride and clomipramine in patients with vascular depression. Chin J Clin Rehab. 2005;9(24):226–228.
12. Ye LX, Wang YD, Wang H, Liang DS. Effect of Paxil and berhomine on post-stroke anxiety-depression and neurological recovery. Chin J Clin Rehab. 2006;10(6):153-155.
13. Li LT, Wang SH, Ge HY, Chen J, Yue SW, Yu M. The beneficial effects of the herbal medicine Free and Easy Wanderer Plus (FEWP) and fluoxetine on post-stroke depression. J Altern Complement Med. 2008;14(14):841–846.
14. Cravello L, Caltagirone C, Spalletta G. The SNRI venlafaxine improves emotional unawareness in patients with post-stroke depression. Hum. Psychopharmacol Clin Exp. 2009;24(4):331–336.
15. Karaiskos D, Tzavellas E, Spengos K, Vassilopoulou S, Paparrigopoulos T. Duloxetine versus citalopram and sertraline in the treatment of post-stroke depression, anxiety, and fatigue. J Neuropsychiatry Clin Neurosci 2012;24(3):349–353.

**Appendix 4: Description of included studies, outcomes**

**Table1. Study characteristic**

| study | Inclusion criteria | Exclusion criteria | | | | profile of prior antidepressant therapy |
| --- | --- | --- | --- | --- | --- | --- |
|  |  | diseases limiting verbal comprehension^a^ | psychiatric illness or substance abuse | CNS diseases | others |  |
| Lipsey 1984 | thromboembolic stroke or intracerebral hemorrhage; moderate or severe depression; with informed consent | severe comprehension deficit | - | - | medical contraindication to nortriptyline | only included patients never being treated with antidepressants |
| Andersen 1994 | acute stroke with depression | decreased consciousness; aphasia; dementia | history of psychiatric illness (except depression more than 1 year earlier) | SAH; Binswanger's disease; previous degenerative or expansive neurological diseases^b^ | - | exclude current antidepressant treatment |
| Torrecillas 1995 | unilateral lesion documented by CT scan and capable of compliance were included. with informed consent. | aphasia >2b/3 according to the Goodglass criteria | alcoholism, drug abuse, any pathological condition capable of resembling a depressive condition | - | - | exclude antidepressant treatment in the 6 months before stroke |
| Miyai 1998 | Stroke rehabilitation inpatients meeting DSM3R criteria for organic mood disorder with depression. | - | History of psychiatric disorders | History of seizure | Arrhythmia; history of myocardial infarction |  |
| Robinson 2000 | acute stroke within 6 months of the onset of the study and age 18–85 | severe comprehension deficit | - | head injury, prior or other brain disease except prior stroke | significant medical illness | patients who taking antidepressants were required to stop the therapy before the study (N=3). |
| Kimura 2000 | acute thromboembolic or intracerebral hemorrhagic infarction who were identified as depressed | decreased consciousness; aphasia; dementia | - | - | - | - |
| Fruehwald 2003 | thromboembolic stroke or intracerebral hemorrhage were verified by CT. moderate or severe depression, as measured by a HAMD>15 | more than mild communication deficit; MMSE<20 | - | previous degenerative or expansive neurological diseases | - | - |
| Kimura 2003 | acute thromboembolic or intracerebral hemorrhagic infarction, and identified as depressed | decreased consciousness; aphasia; dementia | - | - | - | - |
| Rampello 2003 | presence of a recent (<12 months) single ischemic or hemorrhagic stroke, which was documented by CT or MRI; presence of major or minor depression, according to DSM IV criteria, with HDRS>20, BDI> 15 | decreased consciousness; severe aphasia, severe cognitive deficit(MMSE <22) | history of psychiatric illness (except depression for more than 1 year); chronic alcoholism, | previous degenerative or expansive neurological diseases, SAH, Binswanger’s disease | respiratory complications; serious heart diseases;  under anticoagulant treatment | lack of antidepressant treatment within 30 days prior to this study |
| Rampello 2004 | presence of a recent (<12 months) single ischemic or hemorrhagic stroke, (documented by CT or MRI); presence of major or minor depression, according to DSM IV criteria, with HDRS>20 and BDI>15; with informed consent. | severe aphasia, severe cognitive deficit | history of psychiatric illness (other than depression); chronic alcoholism. | previous degenerative or expansive neurologic disease, SAH, Binswanger’s disease | - | lack of antidepressant treatment within 2 weeks prior to this study |
| Huang 2005 | with diagnosis of vascular depression; less than 70 years old | decreased consciousness; dementia; severe mental disorders | history of depression | trauma, tumor, inflammation or demyelination in brain | severe impairment in cardiac function, hepatic function or renal function;  history of drug allergy | - |
| Ye 2006 | post-stroke anxiety and depression, with HAMD-24>21 and HAMA-14>14 | decreased consciousness; understanding problems; | - | - | without stable life signs | - |
| Li 2008 | presence of a recent (<6 weeks) single ischemic or hemorrhagic stroke, documented by CT or MRI; presence of major or minor depression, with a HAMD>20 | severe aphasia; MMSE<23 | a history of psychiatric illness other than depression; chronic alcoholism | epilepsy | abnormal thyroid function | lack of before the enrolment within 2 weeks prior to this study |
| Cravello 2009 | first-ever stroke diagnosis within the last 12 months and diagnosis of post-stroke major depressive-like episode. | severe cognitive impairment （MMSE<12） | history of psychiatric disorders, within 5 years before the stroke | stroke history; degenerative or expansive neurological diseases | atherosclerotic disease, major medical illnesses | - |
| Karaiskos 2012 | diagnosis of the first-ever stroke within the last 12 months, based on clinical history, physical examination, and findings of brain MRI; Diagnosis of PSD, according to DSM-IV. | dementia; Severe cognitive impairment(MMSE<24) | history of a major psychiatric disorder within 5 years before the stroke. | stroke history; degenerative or expansive neurological diseases | atherosclerotic disease or a history of angioplasty or bypass surgery; major medical illness | - |

^a^Diseases limiting verbal comprehension including decreased consciousness, aphasia, dementia, and cognitive disorder.

^b^Degenerative neurological diseases including Parkinson disease and Alzheimer disease; expansive neurological diseases including multiple sclerosis, tumor, hydrocephalus, and amyotrophic lateral sclerosis.

HAMD= Hamilton Depression Rating Scale(or HRSD)；

DSM= Diagnostic and Statistical Manual of Mental Disorders

CT=computed tomography

MRI=magnetic resonance imaging

SAH=subarachnoid hemorrhage

TIA=transient ischemic attack

MMSE= Mini-mental State examination;

BDI=Beck Depression Inventory

**Table 2. Patient characteristics**

| Study | intervention/control  (N) | Mean age  (SD) | Gender  (%, male) | Mean baseline  HAMD (SD) | Hemisphere stroke side  (%, left) | Depression diagnosis  N (%, major depression) | Time since stroke onset |
| --- | --- | --- | --- | --- | --- | --- | --- |
| Lipsey 1984 | Nortriptyline(N=14)  Placebo(N=20) | 62(9)  60(12) | 64  65 | 13.9(0.79)  16.57(0.85) | 50  34 | 7(50%)  12(60%) | 262(437) days  128(190) days |
| Andersen 1994 | Citalopram(N=33)  Placebo(N=33) | 68.2(4.2)  65.8(9.0) | 36  42 | 19.4(3.1)  18.9(2.8) | 36.4  39.4 | NR | 10.6(9.8) weeks  13.2(11.0) weeks |
| Gonzalez 1995 | Fluoxetine(N=26)  Nortriptyline(N=11)  Placebo(N=11) | 66.71(12.60)^a^ | 52 | 23.52  21.48  23.52 | 47.9 | 34(71%) | within 4 weeks |
| Miyai 1998 | Desipramine(N=13)  Trazodone(N=6)  Fluoxetine(N=5) | 73(13)  73(5)  76(7) | 50  33.3  80 | 23(4)  23(5)  21(6) | 58.3  50  20 | NR | 40(7) days  42(5) days  34(8) days |
| Robinson 2000 | Fluoxetine(N=23)  Nortriptyline(N=16)  Placebo(N=17) | 65(14)  64(10)  73(8) | 74  31  53 | 20.4(4.7)  22.5(8.5)  17.5(6.2) | 39.1  37.5  29.4 | 11(48%)  10(63%)  6(35%) | within 6 months |
| Kimura 2000 | Nortrityline(N=21)  Placebo(N=26) | 59.6(9.1)  60.7(11.8) | 47.6  65.4 | 17.38(4.3)  17.92(3.95) | 57.1  42.3 | 14(67%)  19(73%) | 111(137) days  190(243) days |
| Fruehwald 2003 | Fluoxetine(N=28);  Placebo(N=26) | 64.8(13.8)  64.0(14.3) | 46.2  70.8 | 32.8(12.7)  30.3(15.0) | 30.8  50 | NR | 11.0(3.9) days  11.1±3.5 days |
| Kimura 2003 | Nortrityline(N=13)  Placebo(N=14) | 64.8(11.3)  55(15.2) | 46.2  50.0 | 17.0(4.8)  17.4(4.0) | 46.2  35.7 | 6(46%)  11(79%) | 73(101) days  117(159) days |
| Rampello 2003 | Citalopram(N=37)  Reboxetine(N=37) | 73.13(4)  74.71(4.66) | 45.9  48.6 | 22.54(1.87)  22.76(2.02) | 48.6  40.5 | NR | 13.64(5.33) weeks  12.66(4.47) weeks |
| Rampello 2004 | Reboxetine(N=16);  Placebo(N=15) | 77.5(4)  77.26(3.6) | 43.8  46.7 | 24.06(1.52)  24 (1.31) | 56.3  56.3 | NR | 12.06(4.23) weeks  12.26(4.77) weeks |
| Huang 2005 | Fluoxetine(N=30)  Clomipramine(N=30) | 58(6)  NR | 56.7  NR | 21.3(2.64)  20.09(2.1) | NR | NR | NR |
| Ye 2006 | Paroxetine(N=30)  Imipramine(N=30)  Placebo(N=30) | 58.06(8.46)  56.98(11.42)  59.37(9.56) | 73.3  60.0  56.7 | 25.18(7.02)  24.2(9.04)  25.12(5.19) | 60  60  63.3 | NR | NR |
| Lian 2008 | TCM (N=60)  Fluoxetine(N=60)  Placebo(N=30) | 68.5(4.10)  69.2(3.5)  67.8(3.90) | 46.7  41.7  56.7 | 25.2(3.8)  25.5(3.1)  24.3(2.90) | 58.3  51.7  40 | NR | within 6 weeks |
| Cravello 2009 | Fluoxetine(N=25)  Venlafaxine(N=25) | 65.9(12.7)  64.2(14.1) | 36  44 | 19.2(4.4)  17(4.5) | NR | 100% | 146.8(41.5) days  147.6(47.9) days |
| Dimitrios 2012 | Duloxetine(N=20)  Citalopram(N=20)  Sertraline(N=20) | 51.1 (13.4)  54.3 (12.5)  52.4 (11.4) | NR | 24.5 (7.5)  23.7 (6.7)  23.8 (7.3) | NR | NR | within 12 months |

^a^Pooled data from 2 groups: major depression group 67(13); minor depression group 66(12)

TCM= traditional Chinese medicine

NR=not reported

**Table 3. Treatment characteristic**

| study | duration | intervention/control group  (N and maximum daily dose) | | |
| --- | --- | --- | --- | --- |
| Lipsey 1984 | 6 weeks | Nortriptyline  (N=14;100mg/d) | Placebo  (N=20) | - |
| Andersen 1994 | 6 weeks | Citalopram  (N=33;20mg/d) | Placebo  (N=33) | - |
| Torrecillas 1995 | 6 weeks | Fluoxetine  (N=26;20mg/d) | Nortriptyline  (N=11;75mg/d) | Placebo  (N=11;100mg/d) |
| Miyai 1998 | 4 week | Desipramine  (N=13;100mg/d) | Trazodone  (N=6;100mg/d) | Fluoxetine  (N=5;20mg/d) |
| Robinson 2000 | 12 weeks | Fluoxetine  (N=23;40mg/d) | Nortriptyline  (N=16,100mg/d | Placebo  (N=17) |
| Kimura 2000 | 6 or 12 weeks | Nortrityline  (N=21;100mg/d) | Placebo  (N=26) | - |
| Fruehwald 2003 | 12 weeks | Fluoxetine  (N=28; 20mg/d); | Placebo  (N=26) | - |
| Kimura 2003 | 6 or 12 weeks | Nortrityline  (N=13;100mg/d) | Placebo  (N=14) | - |
| Rampello 2003 | 16 weeks | Citalopram  (N=37;20mg/d) | Reboxetine  (N=37;4mg/d) | - |
| Rampello 2004 | 16 weeks | Reboxetine  (N=16;4mg/d); | Placebo  (N=15) | - |
| Huang 2005 | 12 weeks | Fluoxetine  (N=30;20mg/d) | Clomipramine  (N=30;750mg/d) | - |
| Ye 2006 | 12 weeks | Paroxetine  (N=29;20mg/d) | Imipramine  (N=27;150mg/d) | Placebo  (n=27) |
| Li 2008 | 8 weeks | TCM  (N=60;36g/d) | Fluoxetine  (N=60;40mg/d) | Placebo  (N=30;36g/d) |
| Cravello 2009 | 8 weeks | Fluoxetine  (N=25;40mg/d) | Venlafaxine  (N=25;150mg/d) | - |
| Karaiskos 2012 | 3 months | Duloxetine  (N=20;120mg/d) | Citalopram  (N=20;40mg/d) | Sertraline  (N=20;200mg/d) |

**Table 4. Results** **of individual studies**

**4-1 reduction of HAMD score between pre- and post- treatment of antidepressants**

|  | sertraline | | | sertraline | | | fluoxetine | | | paroxetine | | | duloxetine | | | venlafaxine | | | imipramine | | | clomipramine | | | nortriptyline | | | desipramine | | | trazodone | | | reboxetione | | | **TCM** | | | Placebo | | |
| --- | --- | --- | --- | --- | --- | --- | --- | --- | --- | --- | --- | --- | --- | --- | --- | --- | --- | --- | --- | --- | --- | --- | --- | --- | --- | --- | --- | --- | --- | --- | --- | --- | --- | --- | --- | --- | --- | --- | --- | --- | --- | --- |
|  | n | mean | sd | n | mean | sd | n | mean | sd | n | mean | sd | n | mean | sd | n | mean | sd | **n** | **mean** | **sd** | **n** | **mean** | **sd** | **n** | **mean** | **sd** | **n** | **mean** | **sd** | **n** | **mean** | **sd** | n | mean | sd | n | mean | sd | n | mean | sd |
| Lipsey 1984 |  |  |  |  |  |  |  |  |  |  |  |  |  |  |  |  |  |  |  |  |  |  |  |  | **11** | **11.14** | **0.69** |  |  |  |  |  |  |  |  |  |  |  |  | 15 | 6.39 | 1.03 |
| Andersen 1994 | 33 | 8 | 6 |  |  |  |  |  |  |  |  |  |  |  |  |  |  |  |  |  |  |  |  |  |  |  |  |  |  |  |  |  |  |  |  |  |  |  |  | 33 | 4.8 | 4.6 |
| Torrecillas 1995 |  |  |  |  |  |  | 25 | 16.37 | 7.9 |  |  |  |  |  |  |  |  |  |  |  |  |  |  |  | **10** | **14.68** | **0.35** |  |  |  |  |  |  |  |  |  |  |  |  | 10 | 2.11 | 0.36 |
| Miyai 1998 |  |  |  |  |  |  | 4 | 5 | 0.1 |  |  |  |  |  |  |  |  |  |  |  |  |  |  |  |  |  |  | **8** | **6** | **3** | **6** | **8** | **3** |  |  |  |  |  |  |  |  |  |
| Robinson 2000 |  |  |  |  |  |  | 14 | 1.9 | 6.64 |  |  |  |  |  |  |  |  |  |  |  |  |  |  |  | **13** | **13.5** | **7.47** |  |  |  |  |  |  |  |  |  |  |  |  | 13 | 5.3 | 5.6 |
| Kimura 2000 |  |  |  |  |  |  |  |  |  |  |  |  |  |  |  |  |  |  |  |  |  |  |  |  | **18** | **12.05** | **5.53** |  |  |  |  |  |  |  |  |  |  |  |  | 26 | 6.84 | 6.76 |
| Fruehwald 2003 |  |  |  |  |  |  | 26 | 23.3 | 12 |  |  |  |  |  |  |  |  |  |  |  |  |  |  |  |  |  |  |  |  |  |  |  |  |  |  |  |  |  |  | 24 | 19.1 | 15.1 |
| Kimura 2003 |  |  |  |  |  |  |  |  |  |  |  |  |  |  |  |  |  |  |  |  |  |  |  |  | **13** | **10.2** | **8.2** |  |  |  |  |  |  |  |  |  |  |  |  | 14 | 2.8 | 7.4 |
| Rampello 2003^a^ | 34 | 9.3 | 6.38 |  |  |  |  |  |  |  |  |  |  |  |  |  |  |  |  |  |  |  |  |  |  |  |  |  |  |  |  |  |  | 34 | 7.05 | 6.67 |  |  |  |  |  |  |
| Rampello 2004 |  |  |  |  |  |  |  |  |  |  |  |  |  |  |  |  |  |  |  |  |  |  |  |  |  |  |  |  |  |  |  |  |  | 16 | 14.8 | 1.91 |  |  |  | 15 | 1.27 | 2.08 |
| Huang 2005 |  |  |  |  |  |  | 30 | 13.5 | 3.8 |  |  |  |  |  |  |  |  |  |  |  |  | **30** | **12.19** | **3.64** |  |  |  |  |  |  |  |  |  |  |  |  |  |  |  |  |  |  |
| Ye 2006 |  |  |  |  |  |  |  |  |  | 29 | 21.16 | 6.095 |  |  |  |  |  |  | **27** | **19.18** | **7.97** |  |  |  |  |  |  |  |  |  |  |  |  |  |  |  |  |  |  | 27 | 7.8 | 4.591 |
| Li 2008 |  |  |  |  |  |  | 58 | 10.8 | 2.81 |  |  |  |  |  |  |  |  |  |  |  |  |  |  |  |  |  |  |  |  |  |  |  |  |  |  |  | 60 | 10.6 | 3.36 | 28 | 5.6 | 3.5 |
| Cravello 2009 |  |  |  |  |  |  | 25 | 9.07 | 4.17 |  |  |  |  |  |  | 25 | 7.33 | 4.232 |  |  |  |  |  |  |  |  |  |  |  |  |  |  |  |  |  |  |  |  |  |  |  |  |
| Karaiskos 2012 | 20 | 20.6 | 5.96 | 20 | 19.6 | 6.62 |  |  |  |  |  |  | 20 | 21.1 | 6.87 |  |  |  |  |  |  |  |  |  |  |  |  |  |  |  |  |  |  |  |  |  |  |  |  |  |  |  |

^a^The author divided PSD patients into “retarded” and “anxious” groups, and in each group the patients were randomized into citalopram and reboxetine subgroups.

**4-2 r****esponse rate of all antidepressants**

|  | fluoxetine | | paroxetine | | imipramine | | clomipramine | | nortriptyline | | TCM | | placebo | |
| --- | --- | --- | --- | --- | --- | --- | --- | --- | --- | --- | --- | --- | --- | --- |
|  | Response | n | Response | n | Response | n | response | n | response | n | response | n | Response | n |
| Robinson 2000 | 2 | 23 |  |  |  |  |  |  | 10 | 16 |  |  | 4 | 17 |
| Kimura 2000 |  |  |  |  |  |  |  |  | 16 | 21 |  |  | 8 | 26 |
| Fruehwald 2003 | 18 | 26 |  |  |  |  |  |  |  |  |  |  | 18 | 24 |
| Kimura 2003 |  |  |  |  |  |  |  |  | 9 | 13 |  |  | 3 | 14 |
| Huang 2005 | 25 | 30 |  |  |  |  | 25 | 30 |  |  |  |  |  |  |
| Ye 2006 |  |  | 20 | 29 | 18 | 27 |  |  |  |  |  |  | 7 | 27 |
| Li 2008 | 38 | 58 |  |  |  |  |  |  |  |  | 36 | 60 | 6 | 28 |

**4-3 reduction of HAMD score between baseline and 4-week duration of antidepressants**

|  | Citalopram | | | Sertraline | | | Fluoxetine | | | Paroxetine | | | Duloxetine | | | Venlafaxine | | | Imipramine | | | Nortriptyline | | | Desipramine | | | Trazodone | | | Reboxetine | | | TCM | | | PLACEBO | | |
| --- | --- | --- | --- | --- | --- | --- | --- | --- | --- | --- | --- | --- | --- | --- | --- | --- | --- | --- | --- | --- | --- | --- | --- | --- | --- | --- | --- | --- | --- | --- | --- | --- | --- | --- | --- | --- | --- | --- | --- |
|  | n | mean | sd | n | mean | sd | n | mean | sd | n | mean | sd | n | mean | sd | n | mean | sd | n | mean | sd | n | mean | sd | n | mean | sd | n | mean | sd | n | mean | sd | n | mean | sd | n | mean | sd |
| **Lipsey 1984** |  |  |  |  |  |  |  |  |  |  |  |  |  |  |  |  |  |  |  |  |  | 11 | 7.22 | 0.81 |  |  |  |  |  |  |  |  |  |  |  |  | 15 | 5.05 | 1.05 |
| **Andersen 1994** | 33 | 6.9 | 3.77 |  |  |  |  |  |  |  |  |  |  |  |  |  |  |  |  |  |  |  |  |  |  |  |  |  |  |  |  |  |  |  |  |  | 33 | 1.7 | 2.7 |
| **Torrecillas 1995** |  |  |  |  |  |  | 25 | 12.22 | 4.23 |  |  |  |  |  |  |  |  |  |  |  |  | 10 | 9.14 | 0.35 |  |  |  |  |  |  |  |  |  |  |  |  | 10 | 1.18 | 0.36 |
| **Miyai 1998** |  |  |  |  |  |  | 4 | 5 | 0.1 |  |  |  |  |  |  |  |  |  |  |  |  |  |  |  | 8 | 6 | 3 | 6 | 8 | 3 |  |  |  |  |  |  |  |  |  |
| **Robinson 2000** |  |  |  |  |  |  | 14 | 5.23 | 6.64 |  |  |  |  |  |  |  |  |  |  |  |  | 13 | 5.77 | 7.47 |  |  |  |  |  |  |  |  |  |  |  |  | 13 | 2.83 | 5.6 |
| **Kimura 2000** |  |  |  |  |  |  |  |  |  |  |  |  |  |  |  |  |  |  |  |  |  | 21 | 9 | 5.17 |  |  |  |  |  |  |  |  |  |  |  |  | 26 | 4.8 | 6.67 |
| **Fruehwald 2002** |  |  |  |  |  |  | 26 | 18.2 | 9.6 |  |  |  |  |  |  |  |  |  |  |  |  |  |  |  |  |  |  |  |  |  |  |  |  |  |  |  | 24 | 18.6 | 11.9 |
| **Rampello 2004** |  |  |  |  |  |  |  |  |  |  |  |  |  |  |  |  |  |  |  |  |  |  |  |  |  |  |  |  |  |  | 16 | 9.4 | 1.51 |  |  |  | 15 | 0.13 | 1.36 |
| **Ye 2006** |  |  |  |  |  |  |  |  |  | 29 | 11.2 | 8.44 |  |  |  |  |  |  | 27 | 9.37 | 10.29 |  |  |  |  |  |  |  |  |  |  |  |  |  |  |  | 27 | 4.09 | 5.15 |
| **Li 2008** |  |  |  |  |  |  | 58 | 7.08 | 2.81 |  |  |  |  |  |  |  |  |  |  |  |  |  |  |  |  |  |  |  |  |  |  |  |  | 60 | 6.81 | 3.36 | 28 | 2.56 | 3.5 |
| **Cravello 2009** |  |  |  |  |  |  | 25 | 5.7 | 4.17 |  |  |  |  |  |  | 25 | 5.1 | 4.23 |  |  |  |  |  |  |  |  |  |  |  |  |  |  |  |  |  |  |  |  |  |
| **Karaiskos 2012** | 20 | 7.7 | 6.8 | 20 | 6.9 | 7.15 |  |  |  |  |  |  | 20 | 15.8 | 6.63 |  |  |  |  |  |  |  |  |  |  |  |  |  |  |  |  |  |  |  |  |  |  |  |  |

**4-3 reduction of HAMD score between baseline and 8-week duration of antidepressants**

|  | Citalopram | | | Sertraline | | | Fluoxetine | | | Paroxetine | | | Duloxetine | | | Venlafaxine | | | Imipramine | | | Clomipramine | | | Nortriptyline | | | Reboxetione | | | TCM | | | PLACEBO | | |
| --- | --- | --- | --- | --- | --- | --- | --- | --- | --- | --- | --- | --- | --- | --- | --- | --- | --- | --- | --- | --- | --- | --- | --- | --- | --- | --- | --- | --- | --- | --- | --- | --- | --- | --- | --- | --- |
|  | n | mean | sd | n | mean | sd | n | mean | sd | n | mean | sd | n | mean | sd | n | mean | sd | n | mean | sd | n | mean | sd | n | mean | sd | n | mean | sd | n | mean | sd | n | mean | sd |
| Lipsey 1984 |  |  |  |  |  |  |  |  |  |  |  |  |  |  |  |  |  |  |  |  |  |  |  |  | 11 | 11.14 | 0.69 |  |  |  |  |  |  | 15 | 6.39 | 1.03 |
| Andersen 1994 | 33 | 8 | 6 |  |  |  |  |  |  |  |  |  |  |  |  |  |  |  |  |  |  |  |  |  |  |  |  |  |  |  |  |  |  | 33 | 4.8 | 4.6 |
| Torrecillas 1995 |  |  |  |  |  |  | 25 | 16.37 | 4.23 |  |  |  |  |  |  |  |  |  |  |  |  |  |  |  | 10 | 14.68 | 0.35 |  |  |  |  |  |  | 10 | 2.11 | 0.36 |
| Robinson 2000 |  |  |  |  |  |  | 14 | 3.65 | `6.64 |  |  |  |  |  |  |  |  |  |  |  |  |  |  |  | 13 | 9 | 7.47 |  |  |  |  |  |  | 13 | 4.01 | 5.6 |
| Kimura 2000 |  |  |  |  |  |  |  |  |  |  |  |  |  |  |  |  |  |  |  |  |  |  |  |  | 18 | 12.05 | 5.53 |  |  |  |  |  |  | 26 | 6.84 | 6.76 |
| Fruehwald 2002 |  |  |  |  |  |  | 26 | 23.3 | 12 |  |  |  |  |  |  |  |  |  |  |  |  |  |  |  |  |  |  |  |  |  |  |  |  | 24 | 19.1 | 15.1 |
| M. Kimura 2003 |  |  |  |  |  |  |  |  |  |  |  |  |  |  |  |  |  |  |  |  |  |  |  |  | 13 | 10.2 | 8.2 |  |  |  |  |  |  | 14 | 2.8 | 7.4 |
| Rampello 2003 | 34 | 9.31 | 6.38 |  |  |  |  |  |  |  |  |  |  |  |  |  |  |  |  |  |  |  |  |  |  |  |  | 34 | 7.05 | 6.67 |  |  |  |  |  |  |
| Rampello 2004 |  |  |  |  |  |  |  |  |  |  |  |  |  |  |  |  |  |  |  |  |  |  |  |  |  |  |  | 16 | 12.26 | 1.81 |  |  |  | 15 | 0.13 | 1.81 |
| Huang 2005 |  |  |  |  |  |  | 30 | 8.9 | 3.76 |  |  |  |  |  |  |  |  |  |  |  |  | 30 | 6.89 | 4.25 |  |  |  |  |  |  |  |  |  |  |  |  |
| Ye 2006 |  |  |  |  |  |  |  |  |  | 29 | 18.16 | 6.11 |  |  |  |  |  |  | 27 | 16.88 | 8.04 |  |  |  |  |  |  |  |  |  |  |  |  | 27 | 6.1 | 4.52 |
| Li 2008 |  |  |  |  |  |  | 58 | 10.8 | 2.81 |  |  |  |  |  |  |  |  |  |  |  |  |  |  |  |  |  |  |  |  |  | 60 | 10.6 | 3.36 | 28 | 5.6 | 3.5 |
| Cravello 2009 |  |  |  |  |  |  | 25 | 9.07 | 4.17 |  |  |  |  |  |  | 25 | 7.33 | 4.232 |  |  |  |  |  |  |  |  |  |  |  |  |  |  |  |  |  |  |
| Karaiskos 2012 | 20 | 15.6 | 5.83 | 20 | 15.7 | 6.32 |  |  |  |  |  |  | 20 | 21.4 | 6.84 |  |  |  |  |  |  |  |  |  |  |  |  |  |  |  |  |  |  |  |  |  |

**4-5 drop-out rates of all antidepressants**

|  | Citalopram | | Fluoxetine | | Paroxetine | | Imipramine | | Nortriptyline | | Desipramine | | Trazodone | | Reboxetione | | TCM | | PLACEBO | |
| --- | --- | --- | --- | --- | --- | --- | --- | --- | --- | --- | --- | --- | --- | --- | --- | --- | --- | --- | --- | --- |
|  | n | case | n | case | n | case | n | case | n | case | n | case | n | case | n | case | n | case | n | case |
| LIPSEY 1984 |  |  |  |  |  |  |  |  | 17 | 6 |  |  |  |  |  |  |  |  | 22 | 7 |
| Andersen 1994 | 33 | 7 |  |  |  |  |  |  |  |  |  |  |  |  |  |  |  |  | 33 | 2 |
| Torrecillas 1995 |  |  | 26 | 1 |  |  |  |  | 11 | 1 |  |  |  |  |  |  |  |  | 11 | 1 |
| Miyai |  |  | 5 | 1 |  |  |  |  |  |  | 12 | 4 | 6 | 0 |  |  |  |  |  |  |
| Robinson 2000 |  |  | 23 | 9 |  |  |  |  | 16 | 3 |  |  |  |  |  |  |  |  | 17 | 4 |
| Kimura 2000 |  |  |  |  |  |  |  |  | 21 | 3 |  |  |  |  |  |  |  |  | 26 | 0 |
| Fruehwald 2002 |  |  | 28 | 2 |  |  |  |  |  |  |  |  |  |  |  |  |  |  | 26 | 2 |
| Kimura 2003 |  |  |  |  |  |  |  |  | 13 | 1 |  |  |  |  |  |  |  |  | 14 | 0 |
| Rampello 2003 | 37 | 3 |  |  |  |  |  |  |  |  |  |  |  |  | 37 | 3 |  |  |  |  |
| Ye 2006 |  |  |  |  | 30 | 1 | 30 | 3 |  |  |  |  |  |  |  |  |  |  | 30 | 3 |
| Li 2008 |  |  | 60 | 0 |  |  |  |  |  |  |  |  |  |  |  |  | 60 | 2 | 30 | 2 |

**Table 5. Adverse events of individual studies**

| study | Adverse events  (Showed as events n or event rates(%)) | | |
| --- | --- | --- | --- |
|  | Group 1 | Group 2 | Group 3 |
| Lipsey 1984 | nortriptyline: dizziness 1; delirious 3; sedated 1; syncopal 1 | placebo: mania 1; refused interview or unfollowed 3; death 2(heart-failure 1; ICH 1); dizziness 1 |  |
| Andersen 1994 | citalopram: new stroke 3(thromboembolic 1; ICH 1; TIA 1); epilepsy 2; rash 1; death 2(both not because of heart-failure) | placebo: death 2(heart-failure) ; heart-failure 1; acute myocardial infarction 1; new stroke 2(thromboembolic 1;TIA 1); rash 1 |  |
| Torrecillas 1995 | fluoxetine:1 drop out because of side effects; | nortriptyline:1 drop out because of side effects | Placebo: unfollowed |
| Miyai | Desipramine:confusion (two patients), orthostatic hypotension (one pa-  tient), tachycardia (one patient), drug rash (one patient). | Fluoxetine: glaucoma (one patient) |  |
| Robinson 2000 | fluoxetine: gastrointestinal symptoms 3; refused treatment 6; | nortriptyline: medical deterioration 2; refused treatment 1 | placebo: death 1(pulmonary embolus 1); medical deterioration 1; refused treatment 2 |
| Kimura 2000 | NR | | |
| Fruehwald 2003 | fluoxetine: death 1; pulmonary artery embolism 1 | placebo: medical deterioration 1(suicidal 1); dermatological disease 1 |  |
| Kimura 2003 | NR | | |
| Rampello 2003 | citalopram: nausea (3%), vomiting (2%), asthenia and fatigability (4%), opening insomnia (18%), weight increase (12%), and reduction of sexual activity (7%). | reboxetine: dry mouth (19%), constipation (16%), hyperperspiration (16%), drowsiness (5%), urinary wavering or urinary retention (4%), hypotension (7%), and sinusal tachycardia (6%) |  |
| Rampello 2004 | reboxetine: dry mouth (22%), constipation (18%), hyperperspiration (16%), insomnia (4%),drowsiness (3%), urinary wavering or urinary retention (4%), hypotension (8%), and sinusal tachycardia (7%). | placebo: dry mouth (19%), constipation (15%), hyperperspiration (12%), insomnia (5%),drowsiness (5%), hypotension (2%), and sinusal tachycardia (1%). |  |
| Huang 2005 | fluoxetine:8 patients(27%) had adverse events, and 2 of them might be linked to the medication (Nausea, thirsty) | clomipramine:13 patients (43%) had adverse events, 10 of them might be linked to the medication (thirsty, constipation, voiding dysfunction, dizziness and excitation) |  |
| Ye 2006 | paroxetine: unfollowed 1; Nausea 2; | imipramine: refused treatment 3 (because of xerostomia, constipation, blurred vison, cardiovascular side effects) | Placebo: death 1(ICH);unfollowed 2 |
| Li 2008 | TCM:2 Nausea; | fluoxetine: Nausea 6;insomnia 4; second stroke 2 | placebo:3 Nausea; 2 insomnia; 2 aggravated symptoms of depression |
| Cravello 2009 | fluoxetine: insomnia; Nausea, fatigability, cephalalgia, and dizziness(all events were mild) | venlafaxine: headache, insomnia, dry mouth, agitation, sweating, and urinary retention; blood pressure increase(all events were mild) |  |
| Karaiskos 2012 | duloxetine: Nausea 3(15%); somnolence 3(15%); insomnia 1(5%); dizziness 2(10%); dry mouth 2(10%); headache 2(10%) | citalopram: Nausea 4(20%); somnolence 4(20%);dry mouth 2(10%); diarrhea 2(10%); | sertraline: Nausea 5(25%); somnolence 3(15%); insomnia 4(20%); dry mouth 3(15%); diarrhea 4(20%) |

TCM= traditional Chinese medicine

**Appendix 5: Forest plot of direct pairwise meta-analysis for efficacy and safety**

**Figure 1:** **Forest plot of direct pairwise meta-analysis for primary efficacy outcomes (effects after completion of treatments)**

**
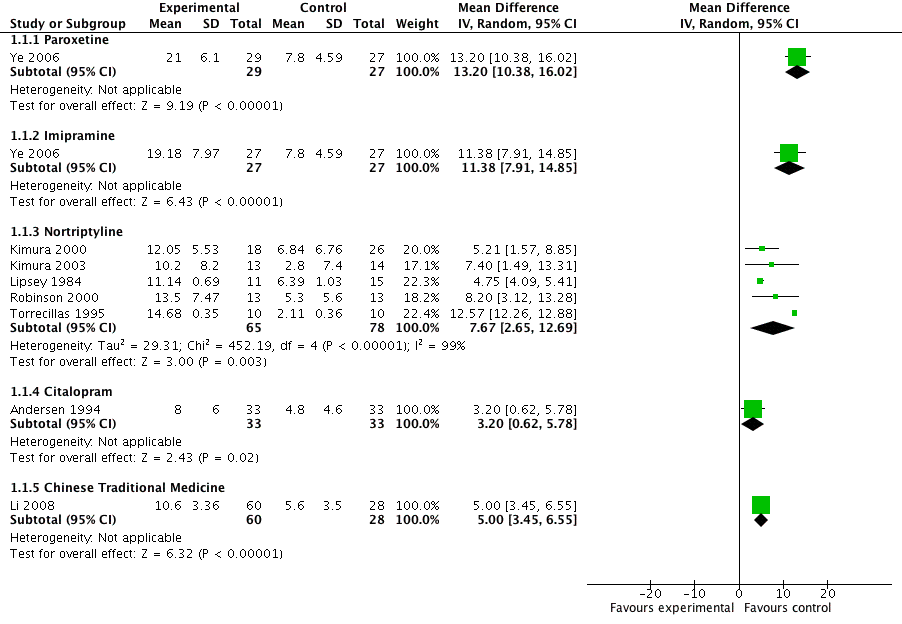
**

**Figure 2: Forest plot of direct pairwise meta-analysis for short-term efficacy outcomes (efficacy of 4-week duration)**

**
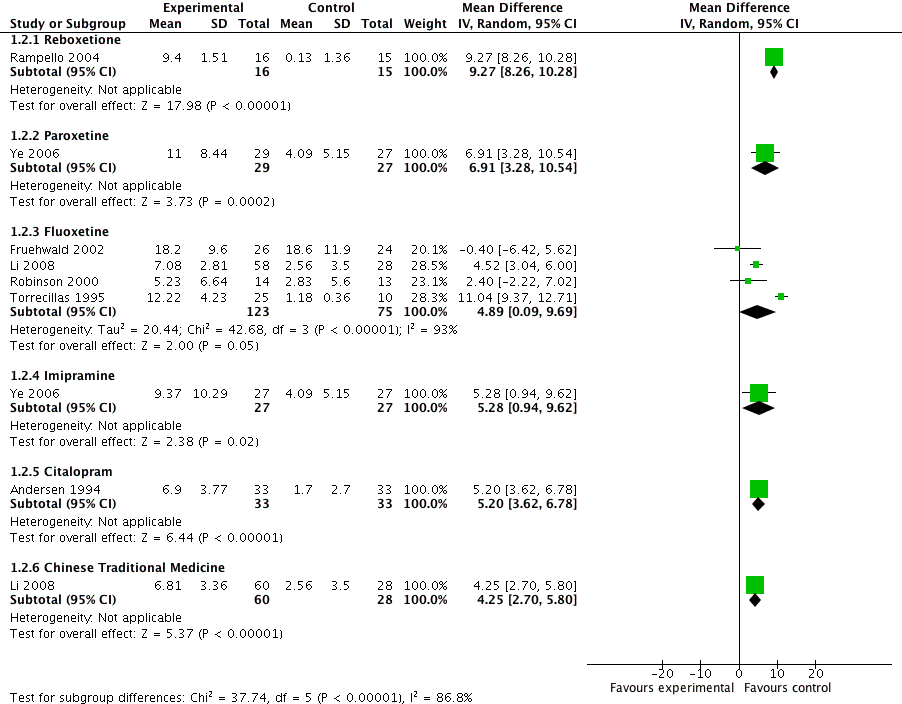
**

**Figure 3: Forest plot of direct pairwise meta-analysis for medium-term efficacy outcomes (efficacy of 8-week duration)**

**
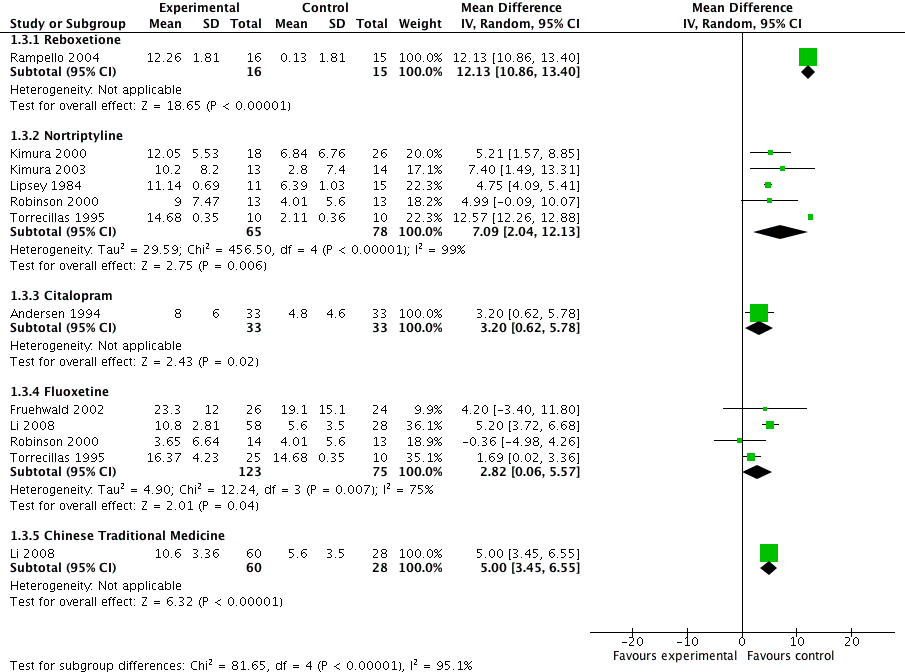
**

**Figure 4: Forest plot of direct pairwise meta-analysis for secondary** **efficacy outcomes of response rate**

**
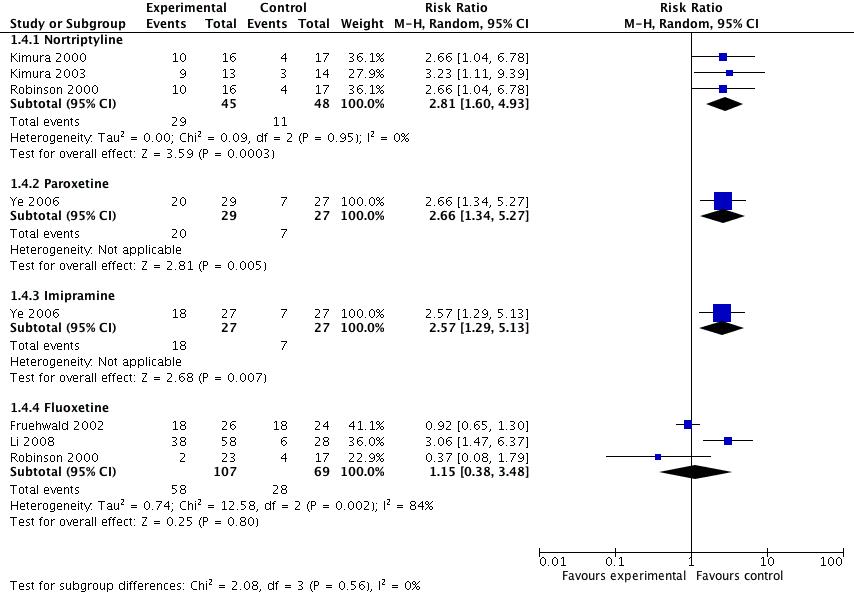
**

**Figure 5: Forest plot of direct pairwise meta-analysis for tolerability outcomes**

**
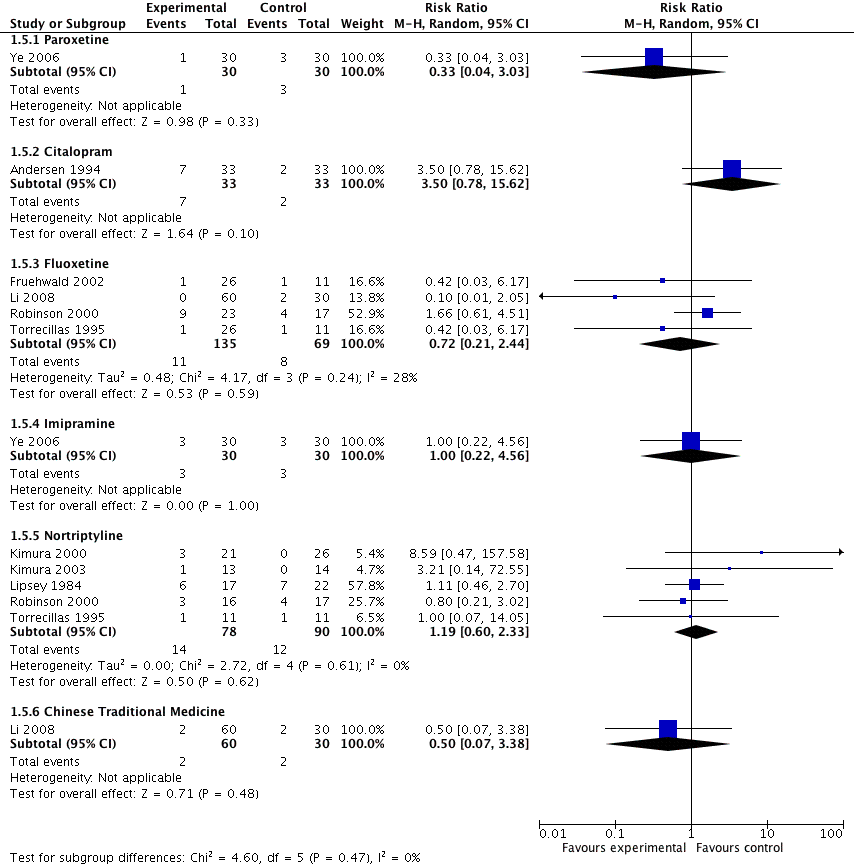
**

**Appendix 6: Results and quality of evidence of network meta-analysis**

**Table 1 Statistically significant results of meta-analysis for end of treatment and quality of evidence.**

| Comparisons | network | pairwise | P-value | Heterogeneity I^2^ | Quality of evidence | Downgraded reason |
| --- | --- | --- | --- | --- | --- | --- |
| Paroxetine VS Imipramine | 2.11(-7.04,11.41) | / | / | / | low | imprecision and indirectness |
| Paroxetine VS Reboxetine | 3.88(-7.60,15.53) | / | / | / | low | imprecision and indirectness |
| Paroxetine VS Trazodone | 5.62(-7.70,18.80) | / | / | / | low | imprecision and indirectness |
| Paroxetine VS Nortriptyline | 5.82(-4.02,15.89) | / | / | / | low | imprecision and indirectness |
| Paroxetine VS Duloxetine | 5.50(-9.51,20.35) | / | / | / | very low | risk of bias and imprecision and indirectness |
| Paroxetine VS Citalopram | 5.85(-5.89,17.83) | / | / | / | low | imprecision and indirectness |
| Paroxetine VS Sertraline | 6.72(-7.69,20.83) | / | / | / | very low | risk of bias and imprecision and indirectness |
| Paroxetine VS Desipramine | 7.58(-5.52,20.79) | / | / | / | low | imprecision and indirectness |
| Paroxetine VS TCM | 8.56(-3.16,20.54) | / | / | / | low | imprecision and indirectness |
| Paroxetine VS Fluoxetine | 8.11(-2.23,18.21) | / | / | / | low | imprecision and indirectness |
| Paroxetine VS Clomipramine | 9.76(-3.48,23.58) | / | / | / | low | imprecision and indirectness |
| Paroxetine VS Venlafaxine | 10.29(-3.00,23.77) | / | / | / | very low | risk of bias and imprecision and indirectness |
| Paroxetine VS Placebo | **13.49(4.36,22.52)** | / | / | / | low | imprecision and indirectness |
| Imipramine VS Reboxetine | 1.80(-9.83,13.60) | / | / | / | low | imprecision and indirectness |
| Imipramine VS Trazodone | 3.41(-9.92,16.67) | / | / | / | low | imprecision and indirectness |
| Imipramine VS Nortriptyline | 3.72(-6.32,13.89) | / | / | / | low | imprecision and indirectness |
| Imipramine VS Duloxetine | 3.52(-11.33,18.35) | / | / | / | low | imprecision and indirectness |
| Imipramine VS Citalopram | 3.88(-7.14,15.53) | / | / | / | low | imprecision and indirectness |
| Imipramine VS Sertraline | 4.75(-10.72,19.17) | / | / | / | very low | risk of bias and imprecision and indirectness |
| Imipramine VS Desipramine | 5.26(-8.11,19.61) | / | / | / | low | imprecision and indirectness |
| Imipramine VS TCM | 4.67(-5.85,15.31) | / | / | / | low | imprecision and indirectness |
| Imipramine VS Fluoxetine | 6.14(-4.35,16.93) | / | / | / | low | imprecision and indirectness |
| Imipramine VS Clomipramine | 7.42(-5.67,20.62) | / | / | / | low | imprecision and indirectness |
| Imipramine VS Venlafaxine | 8.00(-6.45,21.25) | / | / | / | very low | risk of bias and imprecision and indirectness |
| Imipramine VS Placebo | **11.37(2.12,20.33)** | / | / | / | low | imprecision and indirectness |
| Reboxetine VS Nortriptyline | 1.81(-6.44,9.80) | / | / | / | low | imprecision and indirectness |
| Reboxetine VS Duloxetine | 1.91(-9.75,13.65) | / | / | / | very low | risk of bias and imprecision and indirectness |
| Reboxetine VS Citalopram | 2.26(-4.96,9.66) | / | / | / | low | imprecision and indirectness |
| Reboxetine VS Sertraline | 3.13(-8.68,14.70) | / | / | / | very low | risk of bias and imprecision and indirectness |
| Reboxetine VS Desipramine | 3.65(-7.65,15.78) | / | / | / | low | imprecision and indirectness |
| Reboxetine VS TCM | 4.67(-5.85,15.31) | / | / | / | low | imprecision and indirectness |
| Reboxetine VS Fluoxetine | 4.52(-3.98,12.98) | / | / | / | low | imprecision and indirectness |
| Reboxetine VS Clomipramine | 5.80(-6.37,17.39) | / | / | / | low | imprecision and indirectness |
| Reboxetine VS Venlafaxine | 6.38(-5.30,19.02) | / | / | / | very low | risk of bias and imprecision and indirectness |
| Reboxetine VS Placebo | **9.57(2.37,16.58)** | / | / | / | low | imprecision and indirectness |
| Trazodone VS Nortriptyline | 0.16(-9.97,9.52) | / | / | / | low | imprecision and indirectness |
| Trazodone VS Duloxetine | 0.26(-15.33,15.43) | / | / | / | very low | risk of bias and imprecision and indirectness |
| Trazodone VS Citalopram | 0.61(-11.45,12.90) | / | / | / | low | imprecision and indirectness |
| Trazodone VS Sertraline | 1.49(-14.12,16.99) | / | / | / | very low | risk of bias and imprecision and indirectness |
| Trazodone VS Desipramine | 2.00(-7.12,10.06) | / | / | / | low | imprecision and indirectness |
| Trazodone VS TCM | 3.11(-8.64,14.72) | / | / | / | low | imprecision and indirectness |
| Trazodone VS Fluoxetine | 2.88(-4.98,10.99) | / | / | / | low | imprecision and indirectness |
| Trazodone VS Clomipramine | 4.16(-8.64,15.45) | / | / | / | very low | risk of bias and imprecision and indirectness |
| Trazodone VS Venlafaxine | 4.74(-8.45,16.29) | / | / | / | low | imprecision and indirectness |
| Trazodone VS Placebo | 7.88(-1.98,17.74) | / | / | / | low | imprecision and indirectness |
| Nortriptyline VS Duloxetine | 0.10(-12.09,12.41) | / | / | / | very low | risk of bias and imprecision and indirectness |
| Nortriptyline VS Citalopram | 0.45(-7.25,8.39) | / | / | / | low | imprecision and indirectness |
| Nortriptyline VS Sertraline | 1.33(-10.85,13.18) | / | / | / | very low | risk of bias and imprecision and indirectness |
| Nortriptyline VS Desipramine | 1.76(-8.50,11.97) | / | / | / | low | imprecision and indirectness |
| Nortriptyline VS TCM | 2.73(-5.63,11.24) | / | / | / | very low | risk of bias and imprecision and indirectness |
| Nortriptyline VS Fluoxetine | 2.71(-2.27,7.85) | 4.70(-8.31,17.72) | 0.00 | 95.00 | moderate | heterogeneity |
| Nortriptyline VS Clomipramine | 4.00(-6.63,13.75) | / | / | / | very low | risk of bias and imprecision and indirectness |
| Nortriptyline VS Venlafaxine | 4.57(-6.10,14.51) | / | / | / | low | imprecision and indirectness |
| Nortriptyline VS Placebo | **7.61(3.51,11.65)** | **7.67(2.65,12.69)** | 0 | 99.00 | moderate | heterogeneity |
| Duloxetine VS Citalopram | 0.35(-8.89,9.21) | / | / | / | low | risk of bias and indirectness |
| Duloxetine VS Sertraline | 1.23(-8.13,11.16) | / | / | / | very low | risk of bias and imprecision and indirectness |
| Duloxetine VS Desipramine | 2.15(-13.19,17.26) | / | / | / | low | imprecision and indirectness |
| Duloxetine VS TCM | 3.16(-10.85,17.09) | / | / | / | low | imprecision and indirectness |
| Duloxetine VS Fluoxetine | 2.62(-9.22,15.01) | / | / | / | very low | risk of bias and imprecision and indirectness |
| Duloxetine VS Clomipramine | 4.46(-11.01,19.81) | / | / | / | low | imprecision and indirectness |
| Duloxetine VS Venlafaxine | 4.93(-10.35,20.19) | / | / | / | very low | risk of bias and imprecision and indirectness |
| Duloxetine VS Placebo | 8.07(-3.88,19.78) | / | / | / | very low | risk of bias and imprecision and indirectness |
| Citalopram VS Sertraline | 0.87(-8.72,10.56) | / | / | / | very low | risk of bias and imprecision and indirectness |
| Citalopram VS Desipramine | 1.51(-10.52,13.46) | / | / | / | low | imprecision and indirectness |
| Citalopram VS TCM | 2.61(-8.16,12.89) | / | / | / | low | imprecision and indirectness |
| Citalopram VS Fluoxetine | 2.58(-5.87,10.75) | / | / | / | low | imprecision and indirectness |
| Citalopram VS Clomipramine | 3.85(-8.32,15.95) | / | / | / | low | imprecision and indirectness |
| Citalopram VS Venlafaxine | 4.27(-7.94,16.33) | / | / | / | very low | risk of bias and imprecision and indirectness |
| Citalopram VS Placebo | **7.49(0.19,14.50)** | / | / | / | low | imprecision and indirectness |
| Sertraline VS Desipramine | 0.64(-14.65,15.73) | / | / | / | very low | risk of bias and imprecision and indirectness |
| Sertraline VS TCM | 1.70(-12.39,15.24) | / | / | / | very low | risk of bias and imprecision and indirectness |
| Sertraline VS Fluoxetine | 1.61(-10.81,13.78) | / | / | / | very low | risk of bias and imprecision and indirectness |
| Sertraline VS Clomipramine | 2.95(-12.36,18.32) | / | / | / | very low | risk of bias and imprecision and indirectness |
| Sertraline VS Venlafaxine | 3.32(-12.07,18.82) | / | / | / | very low | risk of bias and imprecision and indirectness |
| Sertraline VS Placebo | 6.59(-5.09,18.10) | / | / | / | very low | risk of bias and imprecision and indirectness |
| Desipramine VS TCM | 0.96(-10.42,12.78) | / | / | / | low | imprecision and indirectness |
| Desipramine VS Fluoxetine | 0.88(-7.87,9.44) | / | / | / | low | imprecision and indirectness |
| Desipramine VS Clomipramine | 2.16(-11.01,14.37) | / | / | / | low | imprecision and indirectness |
| Desipramine VS Venlafaxine | 2.73(-10.37,15.40) | / | / | / | very low | risk of bias and imprecision and indirectness |
| Desipramine VS Placebo | 5.93(-3.90,15.78) | / | / | / | low | imprecision and indirectness |
| TCM VS Fluoxetine | 0.01(-7.41,8.73) | / | / | / | low | imprecision and indirectness |
| TCM VS Clomipramine | 1.29(-10.27,13.08) | / | / | / | low | imprecision and indirectness |
| TCM VS Venlafaxine | 1.87(-9.76,14.92) | / | / | / | very low | risk of bias and imprecision and indirectness |
| TCM VS Placebo | 4.85(-2.95,12.64) | / | / | / | very low | risk of bias and imprecision and indirectness |
| Fluoxetine VS Clomipramine | 1.30(-7.54,10.06) | / | / | / | low | imprecision and indirectness |
| Fluoxetine VS Venlafaxine | 1.78(-7.24,10.59) | / | / | / | low | imprecision and indirectness |
| Fluoxetine VS Placebo | **4.91(0.26,9.32)** | 5.31(-1.99,12.62) | 0 | 96.00 | low | inconsistency and heterogeneity |
| Clomipramine VS Venlafaxine | 0.58(-12.61,13.82) | / | / | / | very low | risk of bias and imprecision and indirectness |
| Clomipramine VS Placebo | 3.63(-6.32,13.38) | / | / | / | low | imprecision and indirectness |
| Venlafaxine VS Placebo | 3.13(-6.80,13.04) | / | / | / | very low | risk of bias and imprecision and indirectness |

**Table 2 Statistically significant results of meta-analysis for 4-week duration of treatment and quality of evidence.**

| Comparisons | network | pairwise | P-value | Heterogeneity I^2^ | Quality of evidence | Downgraded reason |
| --- | --- | --- | --- | --- | --- | --- |
| Citalopram VS Sertraline | 0.85(-6.82,8.48) | ／ | ／ | ／ | low | imprecision and indirectness |
| Citalopram VS Fluoxetine | -0.75(-8.97,7.72) | ／ | ／ | ／ | low | imprecision and indirectness |
| Citalopram VS Paroxetine | -0.31(-141.61,118.04) | ／ | ／ | ／ | low | imprecision and indirectness |
| Citalopram VS Duloxetine | **-8.20(-15.84，-0.36)** | ／ | ／ | ／ | low | imprecision and indirectness |
| Citalopram VS Venlafaxine | -0.07(-10.82,10.96) | ／ | ／ | ／ | low | imprecision and indirectness |
| Citalopram VS Imipramine | 1.55(-139.21,122.05) | ／ | ／ | ／ | low | imprecision and indirectness |
| Citalopram VS Nortriptyline | 1.03(-6.71,9.75) | ／ | ／ | ／ | low | imprecision and indirectness |
| Citalopram VS Desipramine | -1.81(-12.97,8.75) | ／ | ／ | ／ | low | imprecision and indirectness |
| Citalopram VS Trazodone | -3.73(-15.05,6.57) | ／ | ／ | ／ | low | imprecision and indirectness |
| Citalopram VS Reboxetine | -3.93(-12.80,0.02) | ／ | ／ | ／ | low | imprecision and indirectness |
| Citalopram VS TCM | 0.24(-8.74,9.48) | ／ | ／ | ／ | low | imprecision and indirectness |
| Citalopram VS Placebo | 5.12(-1.59,11.98) | ／ | ／ | ／ | low | imprecision and indirectness |
| Sertraline VS Fluoxetine | -1.60(-12.59,10.19) | ／ | ／ | ／ | very low | risk of bias and imprecision and indirectness |
| Sertraline VS Paroxetine | -1.17(-140.94,122.02) | ／ | ／ | ／ | low | imprecision and indirectness |
| Sertraline VS Duloxetine | **-9.05(-16.95,-0.75)** | ／ | ／ | ／ | very low | risk of bias and imprecision and indirectness |
| Sertraline VS Venlafaxine | -0.92(-14.52,12.00) | ／ | ／ | ／ | very low | risk of bias and imprecision and indirectness |
| Sertraline VS Imipramine | 0.70(-140.51,123.92) | ／ | ／ | ／ | low | imprecision and indirectness |
| Sertraline VS Nortriptyline | 0.18(-10.66,11.90) | ／ | ／ | ／ | very low | risk of bias and imprecision and indirectness |
| Sertraline VS Desipramine | -2.6(-16.06,10.00) | ／ | ／ | ／ | low | imprecision and indirectness |
| Sertraline VS Trazodone | -4.59(-18.41,8.55) | ／ | ／ | ／ | low | imprecision and indirectness |
| Sertraline VS Reboxetine | -4.79(-16.76,7.98) | ／ | ／ | ／ | low | imprecision and indirectness |
| Sertraline VS TCM | -0.61(-12.44,11.25) | ／ | ／ | ／ | low | imprecision and indirectness |
| Sertraline VS Placebo | 4.32(-6.23,14.82) | ／ | ／ | ／ | low | imprecision and indirectness |
| Fluoxetine VS Paroxetine | 0.44(-138.92,118.44) | ／ | ／ | ／ | low | imprecision and indirectness |
| Fluoxetine VS Duloxetine | -7.45(-18.97,4.21) | ／ | ／ | ／ | very low | risk of bias and imprecision and indirectness |
| Fluoxetine VS Venlafaxine | 0.69(-6.92,8.16) | ／ | ／ | ／ | very low | risk of bias and imprecision and indirectness |
| Fluoxetine VS Imipramine | 2.30(-139.13,130.27) | ／ | ／ | ／ | low | imprecision and indirectness |
| Fluoxetine VS Nortriptyline | 1.78(-2.51,6.19) | **2.85(1.27,4.34)** | 0.40 | 20.00 | low | risk of bias and and inconsistency |
| Fluoxetine VS Desipramine | -1.05(-8.52,5.72) | ／ | ／ | ／ | low | imprecision and indirectness |
| Fluoxetine VS Trazodone | -2.98(-10.48,3.65) | ／ | ／ | ／ | low | imprecision and indirectness |
| Fluoxetine VS Reboxetine | -3.18(-10.57,4.38) | ／ | ／ | ／ | low | imprecision and indirectness |
| Fluoxetine VS TCM | 0.99(-5.20,7.14) | ／ | ／ | ／ | low | imprecision and indirectness |
| Fluoxetine VS Placebo | **6.18(2.07,10.22)** | **4.89(0.09,9.69)** | 0.00 | 93.00 | moderate | heterogeneity |
| Paroxetine VS Duloxetine | -7.89(-129.62,132.43) | ／ | ／ | ／ | low | imprecision and indirectness |
| Paroxetine VS Venlafaxine | 0.25(-121.52,139.35) | ／ | ／ | ／ | low | imprecision and indirectness |
| Paroxetine VS Imipramine | 1.86(-6.74,10.42) | ／ | ／ | ／ | low | imprecision and indirectness |
| Paroxetine VS Nortriptyline | 1.34(-117.12,141.40) | ／ | ／ | ／ | low | imprecision and indirectness |
| Paroxetine VS Desipramine | -1.49(-120.21,139.11) | ／ | ／ | ／ | low | imprecision and indirectness |
| Paroxetine VS Trazodone | -3.42(-123.73,134.41) | ／ | ／ | ／ | low | imprecision and indirectness |
| Paroxetine VS Reboxetine | -3.62(-120.92,138.33) | ／ | ／ | ／ | low | imprecision and indirectness |
| Paroxetine VS TCM | 0.55(-118.51,140.72) | ／ | ／ | ／ | low | imprecision and indirectness |
| Paroxetine VS Placebo | 7.21(-0.64,14.79) | ／ | ／ | ／ | low | imprecision and indirectness |
| Duloxetine VS Venlafaxine | 8.14(-4.86.21.37) | ／ | ／ | ／ | very low | risk of bias and imprecision and indirectness |
| Duloxetine VS Imipramine | 9.75(-131.72,132.14) | ／ | ／ | ／ | low | imprecision and indirectness |
| Duloxetine VS Nortriptyline | 9.23(-2.39,20.49) | ／ | ／ | ／ | very low | risk of bias and imprecision and indirectness |
| Duloxetine VS Desipramine | 6.40(-7.40,18.87) | ／ | ／ | ／ | low | imprecision and indirectness |
| Duloxetine VS Trazodone | 4.47(-9.14,17.73) | ／ | ／ | ／ | low | imprecision and indirectness |
| Duloxetine VS Reboxetine | 4.27(-7.81,16.74) | ／ | ／ | ／ | low | imprecision and indirectness |
| Duloxetine VS TCM | 8.43(-2.58,21.28) | ／ | ／ | ／ | low | imprecision and indirectness |
| Duloxetine VS Placebo | **13.22(2.52,23.48)** | ／ | ／ | ／ | low | imprecision and indirectness |
| Venlafaxine VS Imipramine | 1.61(-138.91,119.44) | ／ | ／ | ／ | low | imprecision and indirectness |
| Venlafaxine VS Nortriptyline | 1.09(-7.61,9.30) | ／ | ／ | ／ | very low | risk of bias and imprecision and indirectness |
| Venlafaxine VS Desipramine | -1.74(-12.17,8.53) | ／ | ／ | ／ | low | imprecision and indirectness |
| Venlafaxine VS Trazodone | -3.67(-14.22,6.03) | ／ | ／ | ／ | low | imprecision and indirectness |
| Venlafaxine VS Reboxetine | -3.87(-14.88,6.76) | ／ | ／ | ／ | low | imprecision and indirectness |
| Venlafaxine VS TCM | 0.30(-8.87,9.97) | ／ | ／ | ／ | low | imprecision and indirectness |
| Venlafaxine VS Placebo | 5.62(-2.76,13.86) | ／ | ／ | ／ | low | imprecision and indirectness |
| Imipramine VS Nortriptyline | -0.52(-119.75,141.62) | ／ | ／ | ／ | low | imprecision and indirectness |
| Imipramine VS Desipramine | -3.35(-124.71,139,73) | ／ | ／ | ／ | low | imprecision and indirectness |
| Imipramine VS Trazodone | -5.28(-126.10,133.91) | ／ | ／ | ／ | low | imprecision and indirectness |
| Imipramine VS Reboxetine | -5.48(-123.61,133.53) | ／ | ／ | ／ | low | imprecision and indirectness |
| Imipramine VS TCM | -1.31(-119.22,140.10) | ／ | ／ | ／ | low | imprecision and indirectness |
| Imipramine VS Placebo | 5.21(-2.91,13.38) | ／ | ／ | ／ | low | imprecision and indirectness |
| Nortriptyline VS Desipramine | -2.83(-11.82,5.82) | ／ | ／ | ／ | low | imprecision and indirectness |
| Nortriptyline VS Trazodone | -4.76(-13.75,3.53) | ／ | ／ | ／ | low | imprecision and indirectness |
| Nortriptyline VS Reboxetine | -4.96(-12.35,3.19) | ／ | ／ | ／ | low | imprecision and indirectness |
| Nortriptyline VS TCM | -0.79(-7.51,6.26) | ／ | ／ | ／ | low | imprecision and indirectness |
| Nortriptyline VS Placebo | **4.28(0.60,7.85)** | **4.44(0.29,8.60)** | 0.00 | 99.00 | moderate | heterogeneity |
| Desipramine VS Trazodone | -1.93(-9.31,4.91) | ／ | ／ | ／ | low | imprecision and indirectness |
| Desipramine VS Reboxetine | -2.13(-12.13,7.85) | ／ | ／ | ／ | low | imprecision and indirectness |
| Desipramine VS TCM | 2.04(-7.30,11.09) | ／ | ／ | ／ | low | imprecision and indirectness |
| Desipramine VS Placebo | 7.19(-1.06,15.26) | ／ | ／ | ／ | low | imprecision and indirectness |
| Trazodone VS Reboxetine | -0.20(-10.33,10.12) | ／ | ／ | ／ | low | imprecision and indirectness |
| Trazodone VS TCM | 3.97(-5.25,14.48) | ／ | ／ | ／ | low | imprecision and indirectness |
| Trazodone VS Placebo | **9.29(0.82,17.48)** | ／ | ／ | ／ | low | imprecision and indirectness |
| Reboxetine VS TCM | 4.17(-5.13,13.12) | ／ | ／ | ／ | low | imprecision and indirectness |
| Reboxetine VS Placebo | **9.25(2.24,16.31)** | ／ | ／ | ／ | low | imprecision and indirectness |
| TCM VS Placebo | 5.14(-1.17,11.56) | ／ | ／ | ／ | low | imprecision and indirectness |

**Table 3 Statistically significant results of meta-analysis for 8-week duration of treatment and quality of evidence.**

| Comparisons | network | pairwise | P-value | Heterogeneity I^2^ | Quality of evidence | Downgraded reason |
| --- | --- | --- | --- | --- | --- | --- |
| Citalopram VS Sertraline | -0.06(-15.49,14.50) | ／ | ／ | ／ | low | imprecision and indirectness |
| Citalopram VS Fluoxetine | 0.93(-7.05,8.87) | ／ | ／ | ／ | very low | risk of bias and imprecision and indirectness |
| Citalopram VS Paroxetine | -5.19(-15.06,5.83) | ／ | ／ | ／ | low | imprecision and indirectness |
| Citalopram VS Duloxetine | -5.59(-14.01,3.28) | ／ | ／ | ／ | low | imprecision and indirectness |
| Citalopram VS Venlafaxine | 2.60(-9.36,14.03) | ／ | ／ | ／ | low | imprecision and indirectness |
| Citalopram VS Imipramine | -4.00(-14.87,6.91) | ／ | ／ | ／ | low | imprecision and indirectness |
| Citalopram VS Clomipramine | 3.06(-8.74,15.00) | ／ | ／ | ／ | low | imprecision and indirectness |
| Citalopram VS Nortriptyline | -0.05(-7.59,7.67) | ／ | ／ | ／ | low | imprecision and indirectness |
| Citalopram VS Reboxetine | -1.79(-8.69,4.93) | ／ | ／ | ／ | low | imprecision and indirectness |
| Citalopram VS TCM | 1.46(-8.65,11.45) | ／ | ／ | ／ | low | imprecision and indirectness |
| Citalopram VS Placebo | **6.92(0.18,13.61)** | ／ | ／ | ／ | low | imprecision and indirectness |
| Sertraline VS Fluoxetine | 0.99(-23.56,19.13) | ／ | ／ | ／ | very low | risk of bias and imprecision and indirectness |
| Sertraline VS Paroxetine | -5.13(-23.56,13.13) | ／ | ／ | ／ | low | imprecision and indirectness |
| Sertraline VS Duloxetine | -5.53(-20.52,9.15) | ／ | ／ | ／ | low | imprecision and indirectness |
| Sertraline VS Venlafaxine | 2.66(-16.87,22.78) | ／ | ／ | ／ | very low | risk of bias and imprecision and indirectness |
| Sertraline VS Imipramine | -3.94(-22.81,14.99) | ／ | ／ | ／ | low | imprecision and indirectness |
| Sertraline VS Clomipramine | 3.13(-15.63,22.27) | ／ | ／ | ／ | low | imprecision and indirectness |
| Sertraline VS Nortriptyline | 0.01(-17.74,17.76) | ／ | ／ | ／ | very low | risk of bias and imprecision and indirectness |
| Sertraline VS Reboxetine | -1.72(-19.10,15.53) | ／ | ／ | ／ | low | imprecision and indirectness |
| Sertraline VS TCM | 1.52(-16.95,20.57) | ／ | ／ | ／ | low | imprecision and indirectness |
| Sertraline VS Placebo | 6.99(-10.99,12.58) | ／ | ／ | ／ | low | imprecision and indirectness |
| Fluoxetine VS Paroxetine | -6.12(-15.20,3.56) | ／ | ／ | ／ | low | imprecision and indirectness |
| Fluoxetine VS Duloxetine | -6.52(-18.00,6.07) | ／ | ／ | ／ | very low | risk of bias and imprecision and indirectness |
| Fluoxetine VS Venlafaxine | 1.67(-14.31,5.05) | ／ | ／ | ／ | very low | risk of bias and imprecision and indirectness |
| Fluoxetine VS Imipramine | -4.93(-14.31,5.05) | ／ | ／ | ／ | low | imprecision and indirectness |
| Fluoxetine VS Clomipramine | 2.13(-5.92,10.98) | ／ | ／ | ／ | low | imprecision and indirectness |
| Fluoxetine VS Nortriptyline | -0.98(-6.09,3.77) | -1.35(-8.19,5.48) | 0.01 | 84.00 | low | risk of bias and hetergenenity |
| Fluoxetine VS Reboxetine | -2.72(-10.24,5.13) | ／ | ／ | ／ | low | imprecision and indirectness |
| Fluoxetine VS TCM | 0.53(-7.07,7.92) | ／ | ／ | ／ | low | imprecision and indirectness |
| Fluoxetine VS Placebo | **5.99(1.48,10.24)** | 6.12(0.50,12.73) | 0.00 | 96.00 | moderate | imprecision |
| Paroxetine VS Duloxetine | -0.40(-13.54,13.55) | ／ | ／ | ／ | low | imprecision and indirectness |
| Paroxetine VS Venlafaxine | 7.79(-5.59,20.84) | ／ | ／ | ／ | low | imprecision and indirectness |
| Paroxetine VS Imipramine | 1.19(-7.27,9.68) | ／ | ／ | ／ | low | imprecision and indirectness |
| Paroxetine VS Clomipramine | 8.25(-5.05,21.05) | ／ | ／ | ／ | low | imprecision and indirectness |
| Paroxetine VS Nortriptyline | 5.14(-2.87,13.82) | ／ | ／ | ／ | low | imprecision and indirectness |
| Paroxetine VS Reboxetine | 3.40(-6.97,13.77) | ／ | ／ | ／ | low | imprecision and indirectness |
| Paroxetine VS TCM | 6.65(-4.47,18.10) | ／ | ／ | ／ | low | imprecision and indirectness |
| Paroxetine VS Placebo | 12.12（3.10，20.10） | ／ | ／ | ／ | low | imprecision and indirectness |
| Duloxetine VS Venlafaxine | 8.19(-6.53,21.58) | ／ | ／ | ／ | very low | risk of bias and imprecision and indirectness |
| Duloxetine VS Imipramine | 1.59(-12.05,15.65) | ／ | ／ | ／ | low | imprecision and indirectness |
| Duloxetine VS Clomipramine | 8.65(-6.99,24.01) | ／ | ／ | ／ | low | imprecision and indirectness |
| Duloxetine VS Nortriptyline | 5.54(-6.55,17.22) | ／ | ／ | ／ | very low | risk of bias and imprecision and indirectness |
| Duloxetine VS Reboxetine | 3.80(-7.43,15.12) | ／ | ／ | ／ | low | imprecision and indirectness |
| Duloxetine VS TCM | 7.05(-6.15,19.99) | ／ | ／ | ／ | low | imprecision and indirectness |
| Duloxetine VS Placebo | **12.52(1.42,23.30)** | ／ | ／ | ／ | low | imprecision and indirectness |
| Venlafaxine VS Imipramine | -6.60(-19.12,5.58) | ／ | ／ | ／ | low | imprecision and indirectness |
| Venlafaxine VS Clomipramine | 0.46(-10.84,13.42) | ／ | ／ | ／ | low | imprecision and indirectness |
| Venlafaxine VS Nortriptyline | -2.65(-12.17,7.04) | ／ | ／ | ／ | very low | risk of bias and imprecision and indirectness |
| Venlafaxine VS Reboxetine | -4.39(-15.62,7.04) | ／ | ／ | ／ | low | imprecision and indirectness |
| Venlafaxine VS TCM | -1.14(-13.46,10.56) | ／ | ／ | ／ | low | imprecision and indirectness |
| Venlafaxine VS Placebo | 4.33(-5.07,13.77) | ／ | ／ | ／ | low | imprecision and indirectness |
| Imipramine VS Clomipramine | 7.07(-5.61,19.68) | ／ | ／ | ／ | low | imprecision and indirectness |
| Imipramine VS Nortriptyline | 3.95(-5.56,13.31) | ／ | ／ | ／ | low | imprecision and indirectness |
| Imipramine VS Reboxetine | 2.22(-9.25,12.99) | ／ | ／ | ／ | low | imprecision and indirectness |
| Imipramine VS TCM | 5.46(-6.30,17.35) | ／ | ／ | ／ | low | imprecision and indirectness |
| Imipramine VS Placebo | 10.93（2.04，19.38） | ／ | ／ | ／ | low | imprecision and indirectness |
| Clomipramine VS Nortriptyline | -3.11(-13.24,6.56) | ／ | ／ | ／ | low | imprecision and indirectness |
| Clomipramine VS Reboxetine | -4.85(-16.45,6.51) | ／ | ／ | ／ | low | imprecision and indirectness |
| Clomipramine VS TCM | -1.60(-14.10,9.91) | ／ | ／ | ／ | low | imprecision and indirectness |
| Clomipramine VS Placebo | 3.86(-5.92,13.15) | ／ | ／ | ／ | low | imprecision and indirectness |
| Nortriptyline VS Reboxetine | -1.74(-9.26,5.70) | ／ | ／ | ／ | low | imprecision and indirectness |
| Nortriptyline VS TCM | 1.51(-7.16,9.72) | ／ | ／ | ／ | low | imprecision and indirectness |
| Nortriptyline VS Placebo | **6.98(2.79,10.56)** | 7.09(2.04,12.13) | 0.00 | 99.00 | moderate | imprecision |
| Reboxetine VS TCM | 3.25(-7.27,13.39) | ／ | ／ | ／ | low | imprecision and indirectness |
| Reboxetine VS Placebo | **8.71(2.13,15.24)** | ／ | ／ | ／ | low | imprecision and indirectness |
| TCM VS Placebo | 5.47(-2.33,13.19) | ／ | ／ | ／ | low | imprecision and indirectness |

**Table 4 Statistically significant results of meta-analysis for response rate and quality of evidence.**

| Comparisons | network | pairwise | P-value | Heterogeneity I^2^ | Quality of evidence | Downgraded reason |
| --- | --- | --- | --- | --- | --- | --- |
| Paroxetine VS Placebo | 6.67(0.99,50.00) | / | / | / | low | imprecision and indirectness |
| Imipramine VS Placebo | 5.88(0.85,50.00) | / | / | / | low | imprecision and indirectness |
| Nortriptyline VS Placebo | **8.33(2.44,25.00)** | 4.90(2.02,11.87) | 0.70 | 0.00 | moderate | imprecision |
| TCM VS Placebo | 0.40(0.04,3.43) | / | / | / | low | imprecision and indirectness |
| Fluoxetine VS Placebo | 0.53(0.11,2.05) | 1.29(0.2,8.44) | 0.00 | 83.00 | moderate | imprecision |
| Clomipramine VS Placebo | 0.55(0.03,6.59) | / | / | / | low | imprecision and indirectness |
| Nortriptyline VS Clomipramine | **16.67(1.12,100.00)** | / | / | / | low | imprecision and indirectness |
| Nortriptyline VS Fluoxetine | **16.67(3.00,100.00)** | / | / | / | very low | risk of bias and imprecision and indirectness |
| Nortriptyline VS TCM | **20.00(2.00,100.00)** | / | / | / | low | imprecision and indirectness |
| Paroxetine VS Fluoxetine | **12.50(1.35,100.00)** | / | / | / | low | imprecision and indirectness |
| Imipramine VS Fluoxetine | **11.11(1.18,100.00)** | / | / | / | low | imprecision and indirectness |

Table 5 Statistically significant results of meta-analysis for drop-out rate and quality of evidence.

| Comparisons | network | pairwise | P-value | Heterogeneity I^2^ | Quality of evidence | Downgraded reason |
| --- | --- | --- | --- | --- | --- | --- |
| Citalopram VS Fluoxetine | 1.45(0.01,9.43) | ／ | ／ | ／ | low | imprecision and indirectness |
| Citalopram VS Paroxetine | 6.66(0.00,9.18) | ／ | ／ | ／ | low | imprecision and indirectness |
| Citalopram VS Imipramine | 6.22(0.00,25.74) | ／ | ／ | ／ | low | imprecision and indirectness |
| Citalopram VS Nortriptyline | 1.23(0.02,15.92) | ／ | ／ | ／ | low | imprecision and indirectness |
| Citalopram VS Desipramine | 5.19(0.00.57.49) | ／ | ／ | ／ | low | imprecision and indirectness |
| Citalopram VS Trazodone | 7.05(0.00,8.49) | ／ | ／ | ／ | low | imprecision and indirectness |
| Citalopram VS Reboxetine | 1.07(0.01,17.79) | ／ | ／ | ／ | low | imprecision and indirectness |
| Citalopram VS TCM | 5.60(0.00,28.24) | ／ | ／ | ／ | low | imprecision and indirectness |
| Citalopram VS Placebo | 5.04(0.01,7.85) | ／ | ／ | ／ | low | imprecision and indirectness |
| Fluoxetine VS Paroxetine | 5.65(0.01,16.72) | ／ | ／ | ／ | low | imprecision and indirectness |
| Fluoxetine VS Imipramine | 1.39(0.03,34.22) | ／ | ／ | ／ | low | imprecision and indirectness |
| Fluoxetine VS Nortriptyline | 3.43(0.31,12.42) | ／ | ／ | ／ | very low | risk of bias and imprecision and indirectness |
| Fluoxetine VS Desipramine | **3.72(0.06,51.41)** | ／ | ／ | ／ | low | imprecision and indirectness |
| Fluoxetine VS Trazodone | 2.43(0.31,12.42) | ／ | ／ | ／ | low | imprecision and indirectness |
| Fluoxetine VS Reboxetine | 2.12(0.04,400.64) | ／ | ／ | ／ | low | imprecision and indirectness |
| Fluoxetine VS TCM | 1.13(0.07,40.98) | ／ | ／ | ／ | low | imprecision and indirectness |
| Fluoxetine VS Placebo | 1.57(0.28,6.19) | 1.02(0.18,4.35) | 0.29 | 0.20 | moderate | imprecision |
| Paroxetine VS Imipramine | 1.66(0.10,132.51) | ／ | ／ | ／ | low | imprecision and indirectness |
| Paroxetine VS Nortriptyline | 3.30(0.12,315.80) | ／ | ／ | ／ | low | imprecision and indirectness |
| Paroxetine VS Desipramine | 3.12(0.03,786.36) | ／ | ／ | ／ | low | imprecision and indirectness |
| Paroxetine VS Trazodone | 5.44(0.00,129.08) | ／ | ／ | ／ | low | imprecision and indirectness |
| Paroxetine VS Reboxetine | 1.05(0.05,2221.90) | ／ | ／ | ／ | low | imprecision and indirectness |
| Paroxetine VS TCM | 1.72(0.03,689.59) | ／ | ／ | ／ | low | imprecision and indirectness |
| Paroxetine VS Placebo | 1.26(0.10,85.37) | ／ | ／ | ／ | low | imprecision and indirectness |
| Imipramine VS Nortriptyline | 1.52(0.06,75.12) | ／ | ／ | ／ | low | imprecision and indirectness |
| Imipramine VS Desipramine | 2.66(0.01,325.70) | ／ | ／ | ／ | low | imprecision and indirectness |
| Imipramine VS Trazodone | 3.27(0.00,32.20) | ／ | ／ | ／ | low | imprecision and indirectness |
| Imipramine VS Reboxetine | 1.00(0.01,792.31) | ／ | ／ | ／ | low | imprecision and indirectness |
| Imipramine VS TCM | 4.65(0.01,132.52) | ／ | ／ | ／ | low | imprecision and indirectness |
| Imipramine VS Placebo | 1.09(0.05,26.20) | ／ | ／ | ／ | low | imprecision and indirectness |
| Nortriptyline VS Desipramine | 2.26(0.02,44.45) | ／ | ／ | ／ | low | imprecision and indirectness |
| Nortriptyline VS Trazodone | 1.15(0.00,6.46) | ／ | ／ | ／ | low | imprecision and indirectness |
| Nortriptyline VS Reboxetine | 6.31(0.02,127.70) | ／ | ／ | ／ | low | imprecision and indirectness |
| Nortriptyline VS TCM | 7.74(0.02,19.20) | ／ | ／ | ／ | low | imprecision and indirectness |
| Nortriptyline VS Placebo | 8.64(0.11,2.61) | 1.47(0.40,7.69) | 0.61 | 0.13 | moderate | imprecision |
| Desipramine VS Trazodone | 7.18(0.00,4.73) | ／ | ／ | ／ | low | imprecision and indirectness |
| Desipramine VS Reboxetine | 1.34(0.00,476,12) | ／ | ／ | ／ | low | imprecision and indirectness |
| Desipramine VS TCM | 7.64(0.01,85.51) | ／ | ／ | ／ | low | imprecision and indirectness |
| Desipramine VS Placebo | 4.55(0.02,31.04) | ／ | ／ | ／ | low | imprecision and indirectness |
| Trazodone VS Reboxetine | 1.39(0.03,64820,77) | ／ | ／ | ／ | low | imprecision and indirectness |
| Trazodone VS TCM | 3.81(0.09,14037.73) | ／ | ／ | ／ | low | imprecision and indirectness |
| Trazodone VS Placebo | 1.81(0.08,6736.10) | ／ | ／ | ／ | low | imprecision and indirectness |
| Reboxetine VS TCM | 8.02(0.00,67.46) | ／ | ／ | ／ | low | imprecision and indirectness |
| Reboxetine VS Placebo | 3.79(0.01,25.39) | ／ | ／ | ／ | low | imprecision and indirectness |
| TCM VS Placebo | 1.08(0.03,18.69) | ／ | ／ | ／ | low | imprecision and indirectness |

**Appendix 7. Statistically significant results of network meta-analysis for sensitive analysis.**

| Comparisons | network |
| --- | --- |
| Citalopram VS Sertraline | 0.98(-8.15,10.77) |
| Citalopram VS Fluoxetine | 9.59(-4.57,22.38) |
| Citalopram VS Paroxetine | 1.68(-14.31,16.32) |
| Citalopram VS Duloxetine | -0.67(-10.87,9.13) |
| Citalopram VS Imipramine | 3.82(-11.60,18.15) |
| Citalopram VS Clomipramine | 10.74(-5.35,25.61) |
| Citalopram VS Nortriptyline | 4.97(-5.35,25.61) |
| Citalopram VS Desipramine | 8.82(-7.89,25.24) |
| Citalopram VS Trazodone | 6.72(-9.93,22.03) |
| Citalopram VS Reboxetine | 1.88(-6.92,22.03) |
| Citalopram VS TCM | 10.00(-5.10,24.52) |
| Citalopram VS Placebo | 15.30(2.76,27.46) |
| Sertraline VS Fluoxetine | 8.60(-7.90,25.11) |
| Sertraline VS Paroxetine | 0.70(-17.22,18.00) |
| Sertraline VS Duloxetine | -1.66(-11.40,7.44) |
| Sertraline VS Imipramine | 2.83(-14.78,20.08) |
| Sertraline VS Clomipramine | 9.76(-8.16,29.01) |
| Sertraline VS Nortriptyline | 3.98(-12.38,19.36) |
| Sertraline VS Desipramine | 7.83(-11.53,26.77) |
| Sertraline VS Trazodone | 5.73(-13.27,23.52) |
| Sertraline VS Reboxetine | 0.89(-12.41,13.40) |
| Sertraline VS TCM | 9.02(-8.39,26.55) |
| Sertraline VS Placebo | 14.30(-1.33,30.14) |
| Fluoxetine VS Paroxetine | -7.91(-18.24,2.22) |
| Fluoxetine VS Duloxetine | -10.30(-26.43,7.31) |
| Fluoxetine VS Imipramine | -5.77(-15.88,3.64) |
| Fluoxetine VS Clomipramine | 1.15(-8.01,3.64) |
| Fluoxetine VS Nortriptyline | -4.62(-11.12,1.24) |
| Fluoxetine VS Desipramine | -0.77(-9.20,8.35) |
| Fluoxetine VS Trazodone | -2.87(-11.12,1.24) |
| Fluoxetine VS Reboxetine | -7.71(-17.78,2.07) |
| Fluoxetine VS TCM | 0.42(-7.32,8.10) |
| Fluoxetine VS Placebo | **5.70(0.98,10.20)** |
| Paroxetine VS Duloxetine | -2.36(-20.25,18.08) |
| Paroxetine VS Imipramine | 2.14(-6.82,11.88) |
| Paroxetine VS Clomipramine | 9.06(-4.20,22.19) |
| Paroxetine VS Nortriptyline | 3.29(-7.72,14.48) |
| Paroxetine VS Desipramine | 7.14(-5.76,20.99) |
| Paroxetine VS Trazodone | 5.04(-8.13,18.33) |
| Paroxetine VS Reboxetine | 0.19(-12.00,12.96) |
| Paroxetine VS TCM | 8.32(-2.86,19.98) |
| Paroxetine VS Placebo | 13.60(4.58,22.53) |
| Duloxetine VS Imipramine | 4.50(-14.44,23.15) |
| Duloxetine VS Clomipramine | 11.40(-7.37,31.15) |
| Duloxetine VS Nortriptyline | 5.64(-12.72,22.80) |
| Duloxetine VS Desipramine | 9.49(-10.22,28.83) |
| Duloxetine VS Trazodone | 7.39(-12.20,25.48) |
| Duloxetine VS Reboxetine | 2.55(-10.96,14.65) |
| Duloxetine VS TCM | 10.70(-7.97,27.24) |
| Duloxetine VS Placebo | 16.00(-1.55,31.41) |
| Imipramine VS Clomipramine | 6.92(-5.71,20.70) |
| Imipramine VS Nortriptyline | 1.15(-9.61,12.32) |
| Imipramine VS Desipramine | 5.00(-7.62,18.86) |
| Imipramine VS Trazodone | 2.90(-9.99,16.09) |
| Imipramine VS Reboxetine | -1.95(-14.71,10.31) |
| Imipramine VS TCM | 6.18(-5.04,19.00) |
| Imipramine VS Placebo | **11.5(2.57,20.17)** |
| Clomipramine VS Nortriptyline | -5.77(-16.37,4.58) |
| Clomipramine VS Desipramine | -1.92(-14.79,10.40) |
| Clomipramine VS Trazodone | -4.02(-16.59,8.37) |
| Clomipramine VS Reboxetine | -8.87(-23.00,4.44) |
| Clomipramine VS TCM | -0.74(-12.32,10.68) |
| Clomipramine VS Placebo | 4.54(-5.45,14.40) |
| Nortriptyline VS Desipramine | 3.85(-6.55,15.19) |
| Nortriptyline VS Trazodone | 1.75(-8.66,12.90) |
| Nortriptyline VS Reboxetine | -3.09(-13.63,8.07) |
| Nortriptyline VS TCM | 5.04(-4.07,13.94) |
| Nortriptyline VS Placebo | **10.30(4.12,16.79)** |
| Desipramine VS Trazodone | -2.10(-10.97,7.44) |
| Desipramine VS Reboxetine | -6.94(-20.26,6.63) |
| Desipramine VS TCM | 1.19(-11.5,12.61) |
| Desipramine VS Placebo | 6.47(-3.38,15.55) |
| Trazodone VS Reboxetine | -4.84(-17.86,8.67) |
| Trazodone VS TCM | 3.29(-9.33,14.95) |
| Trazodone VS Placebo | 8.57(-1.41,18.52) |
| Reboxetine VS TCM | 8.13(-3.92,20.29) |
| Reboxetine VS Placebo | **13.4(4.48,22.14)** |
| TCM VS Placebo | 5.28(-2.35,12.68) |

**Appendix 8: Risk of bias assessments within studies**

We used an updated “Risk of bias” tool from the Cochrane Collaboration recommends. This tool addresses seven specific bias domains including methods for generating the random sequence, allocation concealment, blinding of participants and investigators, blinding of outcome assessment, incompleteness of outcome data and selective outcome reporting. Each item is adjudicated within each study and the results are represented in a risk of bias table. We considered allocation concealment adequate if the investigators responsible for patient selection were unable to suspect before allocation which treatment was next. We considered blinding of patients adequate if interventions were described as indistinguishable, or if double-dummy technique was used. We considered blinding of therapists adequate if it was explicitly mentioned in the text that therapists were blinded. We considered incomplete outcome data if it excluded at least one of the randomly assigned patients from the analysis.

Publication bias and selective might affect interventions and comparisons in different ways depending on the clinical context in the network meta-analysis. Using methodology from ecology, attempts have been made to associate the possibility of selection bias with asymmetry measures of the network. Funnel plot asymmetry can be caused by the association between sample size, heterogeneity, and the probability of publication. Sponsorships bias may reflect subtle or less subtle differences in the study designs or the conduct of a trial that only supports the preferred strategy.

(A)Risk of bias graph: review authors' judgements about each risk of bias item presented as percentages across all included studies.


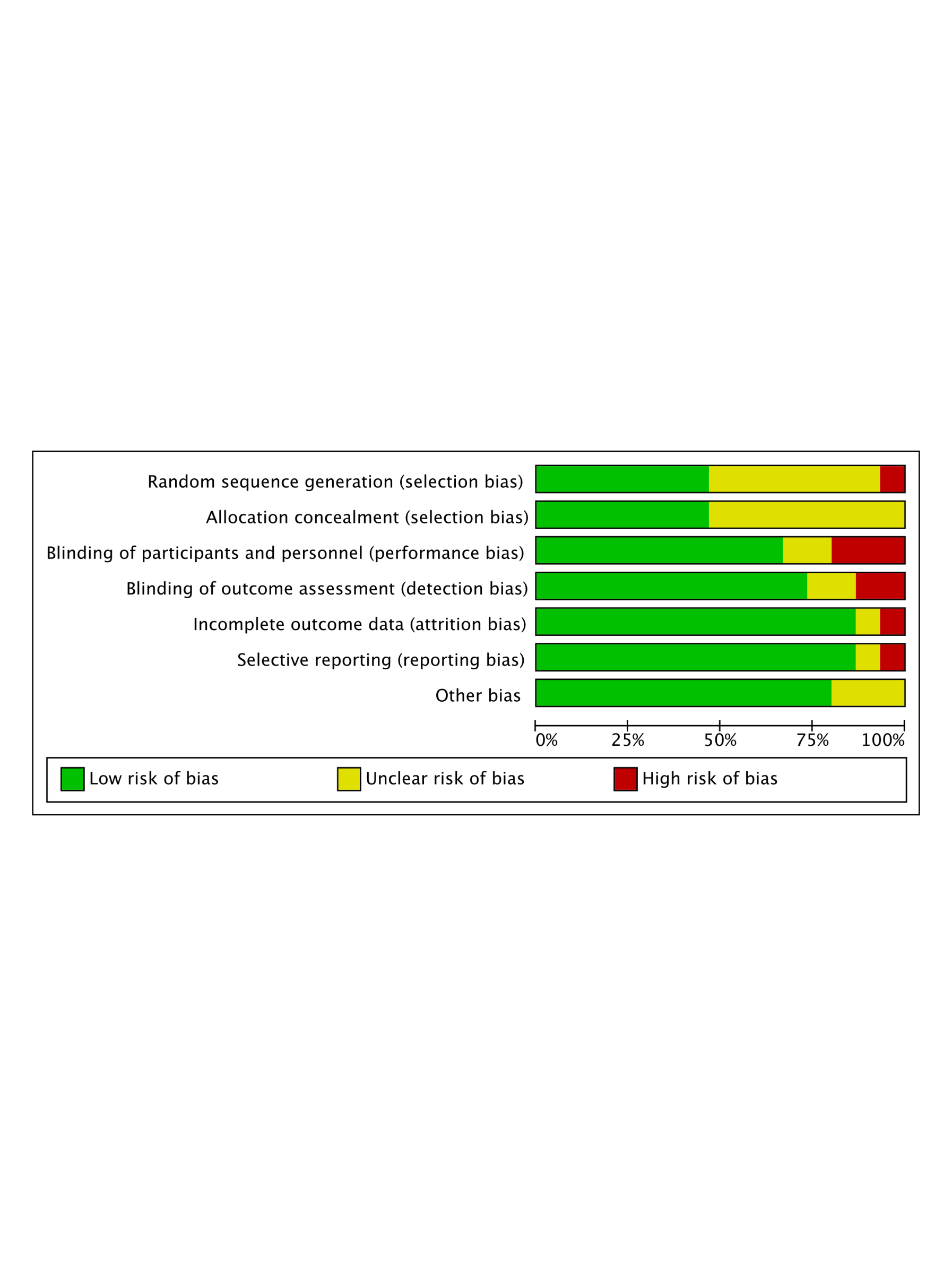


(B)Study-level risk of bias

**
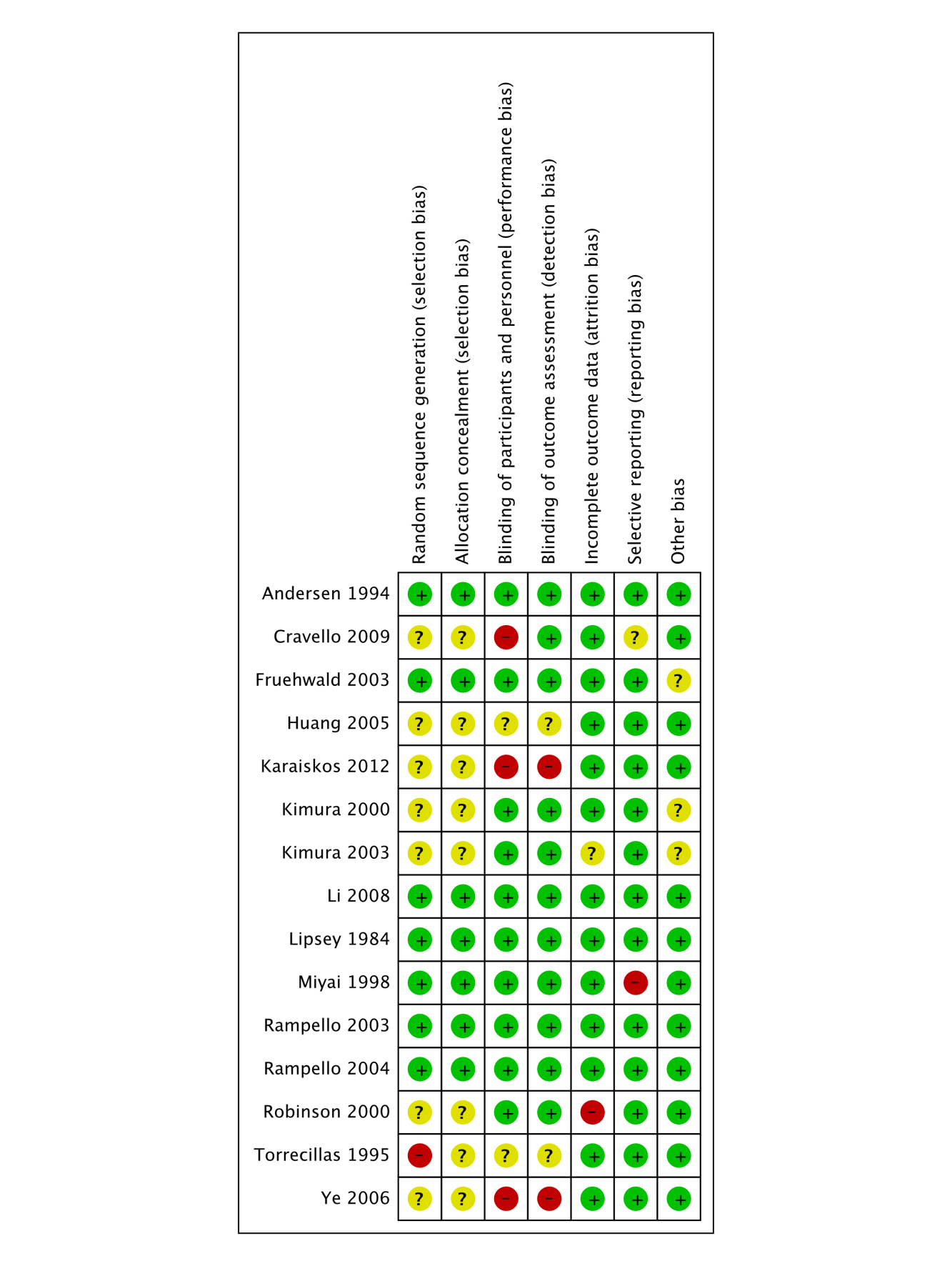
**

Small-study effects assessed via comparison -adjusted network funnel plots. In this presentation, all studies are centered on the summary effect estimate of their respective comparisons [μXY (logOR for present study)] which is represented by the vertical red line. Individual study-level effect size is represented by yiXY [where X and Y are two study agents]. The green line represents linear regression of the comparison specific differences yi - μXY on the standard error of yi. Outer dotted lines indicate the triangular region within which 95% of studies are expected to lie in the absence of both biases and heterogeneity (logOR ± 1.96*standard error). Please note that this is drawn only for comparisons with 2 or more studies.

**Table1. Risk of bias and sponsorship of included studies**

| Study | Random sequence generation | Allocation concealment | Participant blinding | Investigator binding | Incomplete  outcome data | Selective reporting | Other source of bias | Industry sponsorship |
| --- | --- | --- | --- | --- | --- | --- | --- | --- |
| Lipsey 1984 | Low | Low | Low | Low | Low | Low | Low | Low |
| Andersen 1994 | Low | Low | Low | Low | low | Low | Low | Low |
| Torrecillas 1995 | High^a^ | Unclear | Unclear | Unclear | Low | Low | Low | Unclear |
| Miyai 1998 | Low | Low | Low | Low | Low | Low | High^b^ | Low |
| Robinson 2000 | Unclear | Unclear | Low | Low | High^c^ | Low | Low | Unclear |
| Kimura 2000 | Unclear | Unclear | Low | Low | Low | Low | Unclear | Low |
| Fruehwald 2003 | Low | Low | Low | Low | Low | Low | Low | Unclear |
| Kimura 2003 | Unclear | Unclear | Low | Low | Unclear | Low | Unclear | Low |
| Rampello 2003 | Low | Low | Low | Low | Low | Low | Low | Unclear |
| Rampello 2004 | Low | Low | Low | Low | Low | Low | Low | Unclear |
| Huang 2005 | Unclear | Unclear | Unclear | Unclear | Low | Low | Low | Unclear |
| Ye 2006 | Unclear | Unclear | Low | Low | Low | Unclear | Low | Unclear |
| Li 2008 | Low | Low | Low | Low | Low | Low | Low | Low |
| Cravello 2009 | Unclear | Unclear | High | Low | Low | Unclear | Low | Unclear |
| Karaiskos 2012 | Unclear | Unclear | High | High | Low | Low | Low | Low |

^a^the intervention group and placebo group were divided randomly, but the 2 intervention group were divided according to their medical condition.

^b^the sample size were small

^c^the dropout rate was significantly greater in the fluoxetine group than in the nortriptyline and placebo groups (χ2=4.10, df=1, p=0.04)
